# Ionothermal Synthesis of Cadmium Coordination Polymers: Ionic Liquid Effects on the Synthesis, Structural, and Thermal Characterization

**DOI:** 10.3390/molecules24224059

**Published:** 2019-11-09

**Authors:** Iñigo PerezF, Edurne S. Larrea, Begoña Bazán, Gotzone Barandika, M. Karmele Urtiaga, Maria I. Arriortua

**Affiliations:** 1 Dpto. Mineralogía y Petrología, Universidad del País Vasco, UPV/EHU, Sarriena s/n, 48940 Leioa, Spain; inigo.perezf@ehu.eus (I.P.); bego.bazan@ehu.eus (B.B.); karmele.urtiaga@ehu.eus (M.K.U.); maribel.arriortua@ehu.eus (M.I.A.); 2 BCMaterials (Basque Center for Materials, Applications & Nanostructures), UPV/EHU Scientific Park, Martina Casiano Building, 3th floor, Sarriena s/n, 48940 Leioa, Spain; gotzone.barandika@ehu.eus; 3 Dpto. Química Inorgánica, Universidad del País Vasco, UPV/EHU, Sarriena s/n, 48940 Leioa, Spain

**Keywords:** Cadmium coordination polymers, ionothermal synthesis, crystal structure determination

## Abstract

Ionothermal synthesis is a little used method for the preparation of coordination polymers. By this method, two cadmium compounds were synthesized, **1**, with formula Cd_3_(ox)F_2_(Ina)_2_ (Ina = isonicotinate) and **2**, Cd(NO_3_)_2_(4,4′-Bpy) (4,4′-Bpy = 4,4′-Bipyridine). The modification of the reaction conditions has allowed to obtain **2** as a pure phase. The structure of both compounds was determined by a single-crystal X-ray diffraction. Compound **1** is isostructural to the previously reported Cd_2_Zn(ox)(OH)_2_(Ina)_2_. It crystallizes in the monoclinic space group P*2*_1_*/n* and present a three-dimensional (3D) network, built-up from [Cd_3_(ox)F_2_]_n_^2n+^ layers, linked by isonicotinate ligands. Crystals of **2** are formed by twins of two components which are rotated ca. 180° to each other. This compound crystallizes in the triclinic P*-1* space group and its structure can be describe as a two-dimensional (2D) 4 connected *‘sql’* net. The layers are composed by [Cd(NO_3_)_2_]_n_ chains linked through 4,4′-Bpy ligands, and are pillared along the [011] direction. The thermal decomposition of **2** was studied by thermogravimetric and thermodiffractiometric techniques. The compound decomposes gradually starting from 160 °C, and due to heating, the structure suffers slight reversible changes in the bond distances and angles.

## 1. Introduction

The need for new materials capable of responding to current social challenges has allowed the advancement of many fields related to Materials Science in the last decades. The enormous boom that coordination chemistry has experienced since the appearance of metal-organic frameworks (MOFs) is worth mentioning [1] including, porous coordination polymers with unique features for numerous applications [2,3]. The main advantages of MOFs are their ordered structures, high thermal stability, tunable chemical functionality, and ultra-high porosity.

In general, these kinds of compounds are prepared by hydro(solvo)thermal methods, using water (hydrothermal) or organic solvents and mixtures (solvothermal) over their boiling point into sealed reaction containers. The resulting reaction products are usually MOF crystals or microcrystalline powders. Exploring new synthetic methods has allowed existing MOFs to be obtained in other presentations, for example hydrogels or monoliths [4,5], and to scale the synthesis for industrial applications [6], and also obtaining new compounds with different structural and chemical features to those previously reported [7].

In this sense, ionothermal synthesis has been demonstrated to be a proper synthetic method, not only to synthesize known MOFs [8,9,10], but also to functionalize them with ionic liquids and obtain new coordination polymers [11,12,13,14,15]. Ionothermal synthesis consists of replacing water and/or organic solvents by ionic liquids (ILs). ILs are salts formed by organic cations and with melting points below 100 °C [16]. They have wide liquid range, low melting points, and almost negligible vapor pressure. These properties, in combination with their ability for tailoring size, shape, and functionality, differ from conventional solvents [17]. Therefore, the use of ILs in synthesis is a very interesting approach in obtaining compounds, that are not obtainable by using molecular solvents. ILs are also considered to be sustainable solvents [18].

Recently, Vaid et al. [15] have reviewed the effects of ionic liquids in the ionothermal synthesis of MOFs. They have observed that ILs act as structure directing agents in synthesis, either by incorporating part of the IL to the structure (cations as charge balancing and anions coordinated to the metal centers) or not. Even in this case, the IL has a structure-directing effect, as can be observed by the formation of different polymorphs when using different ILs [19,20]. In addition, the use of halide-containing anions (BF_4_^–^, PF_6_^–^…) can lead to the incorporation of the halides to the structure of the coordination polymer [12]. Moreover, in some cases, in situ ligand synthesis occurs during the reaction [21].

In the present work, we have studied the ionothermal synthesis of cadmium coordination polymers by using 1-Butyl-3-methylimidazolium tetrafluoroborate ([Bmim][BF_4_]) and two different ligands, 4-methylpyridine and 4,4′-bipyridine. We have obtained two coordination polymers, the first one, Cd_3_(ox)F_2_(Ina)_2_ (Ina = isonicotinate), isostructural to a previously reported compound, Cd_2_Zn(ox)(OH)_2_(Ina)_2_ [22], and the second one, a novel cadmium-bipyridine coordination polymer, with chemical formula Cd(NO_3_)_2_(4,4′-Bpy) (4,4′-Bpy = 4,4′-Bipyridine) and two-dimensional structure.

## 2. Results and Discussion

### 2.1. Ionothermal Synthesis

For the preparation of cadmium coordination polymers, Cd(NO_3_)_2_·4H_2_O, 4-methylpyridine and 4,4′-Bipyridine were used as reagents in combination with [Bmim][BF_4_] to obtain two coordination polymers: **1**, with formula Cd_3_(ox)F_2_(Ina)_2_ (ox = oxalate, Ina = isonicotinate); and **2**, with formula Cd(NO_3_)_2_(4,4′-Bpy) (4,4′-Bpy = 4,4′-Bipyridine). The starting reaction conditions of temperature and time were decided taking into account ionothermal reactions from the literature. However, in order to obtain pure samples, both temperature and reaction-time were modified. The reactants molar ratio was also modified with the same aim.

In the synthesis of **1**, several chemical transformations of the reactants take place during the heating process. On the one hand, there is an in situ oxidation of the 4-methylpyridine reactant. This reaction, which typically takes place in the presence of a catalyst and an oxidizing agent [23], could take place due to the synergistic effect of Cd^2+^, NO_3_^–^ and [Bmim][BF_4_], as it has been previously described by Xie et al. [12]. On the other hand, there is an in situ formation of oxalate ligands, which could be the product of the hydrolysis of 4-methylpyridine, as it has been previously observed in solvothermal conditions for pyridine derivatives [24]. Finally, there is an in situ hydrolysis of the BF_4_^–^ anion, which gives rise to F^–^ ions, which coordinates to Cd^2+^. This phenomenon has also been described by Xie et al. [12]. Therefore, in this reaction, the ionic liquid plays more roles than the solvent, being a promoter of the ligand oxidation reaction, as well as a source of fluorine. All these in situ transformations seem to be favored by the increase of the reaction temperature, because at higher temperatures, the impurities formed during the reaction almost disappear (see Figure S1, in the supplementary material).

In the synthesis of **2**, 4,4′-Bpy was added to the reaction apart from 4-methylpyridine with the aim of obtaining a compound with both ligands. However, only 4,4′-Bpy is present in **2** structure. The role of the [Bmim][BF_4_] is not so clear in this case, because the compound does not incorporate nor the 4-methylpyridine reactant neither the isonicotinate derivative. In this case, only the 4,4′-Bpy ligand takes part of the crystal structure. On the other hand, the NO_3_^–^ are also incorporated in the structure coordinated to cadmium. The formation of **2** is favored by the decrease of the reaction temperature. Therefore, the side reactions, such as 4-methypyridine oxidation or BF_4_^–^ hydrolysis, may interfere in the formation of **2,** giving rise to impurities (see Figure S2, in the supplementary material). In addition, the removal of 4-methylpyridine from the synthesis do not affect the reaction result.

### 2.2. Crystal Structure

Compound **1**, with chemical formula Cd_3_(ox)F_2_(Ina)_2_, crystallizes in the monoclinic space group P*2*_1_*/n*, and is isostructural to a previously reported mixed-metal coordination polymer with chemical formula Cd_2_Zn(ox)(OH)_2_(Ina)_2_ [22]. The same compound was also previously described by Xie et al. [12], but no crystallographic data were provided by the authors.

**Figure 1:**
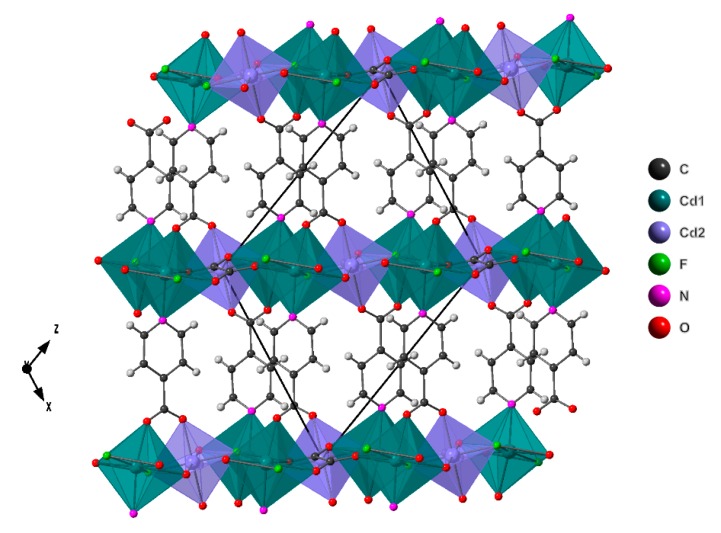
Crystal structure representation of compound **1**.

The compound presents a three-dimensional crystal structure built up from [Cd_3_(ox)F_2_]_n_^2n+^ layers linked by isonicotinate bridging ligands (Figure 1).

There are two crystallographic independent Cd atoms, Cd(1), equivalent to the cadmium atoms of the Zhou et al. structure, and Cd (2), equivalent to the zinc atoms of the isostructural compound. Cd(1) has a coordination number of seven and the coordination environment can be described as a distorted pentagonal bipyramid. The distortion of the coordination environment was calculated with Shape program [25] by the Continuous Shape Measure method. The value of S(PBP) = 0.628 was obtained for the Cd(1) environment, which means a slight deviation from the ideal pentagonal bipyramid [25,26]. On the other hand, Cd(2) shows a coordination of six in an slightly distorted octahedron. The Continuous Shape measure for the polyhedral gives a distortion value from the ideal octahedral of S(O_h_) = 0.635,indicating a slight distorsion [27,28]. The Cd(1) atom coordinates in the equatorial plane to two μ_3_- bridged fluorine atoms and to three oxygen atoms of two different oxalate ligands, while in the axial position it coordinates to one nitrogen atom of one isonicotinate ligand and to one carboxylic oxygen atom, O(2), of another isonicotinate ligand. Regarding the Cd(2) atoms, are linked to two μ_3_- bridged fluorine atoms, and two oxygen atoms of two oxalate molecules in the equatorial plane, while in the axial positions, Cd(2) is coordinated to carboxylic oxygen atom O(1) of two different isonicotinate ligands.

**Figure 2:**
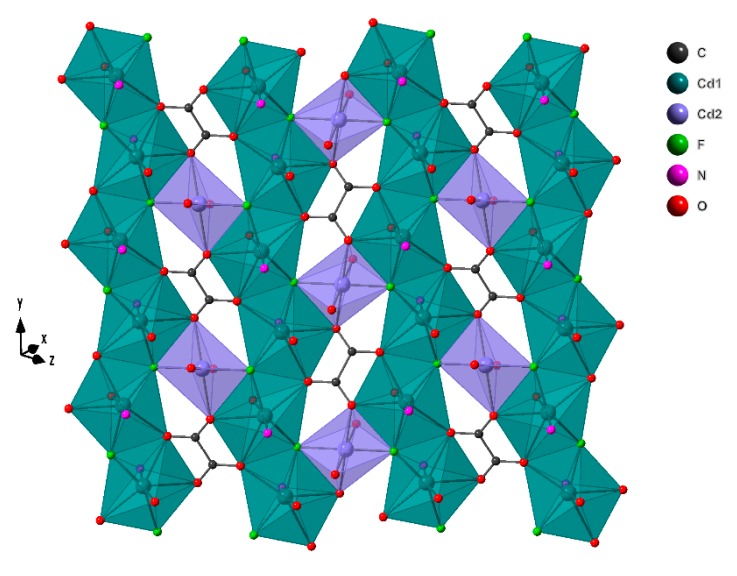
[Cd_3_(ox)F_2_]_n_^2n+^ layer present in the crystal structure of compound **1**.

The [Cd_3_(ox)F_2_]_n_^2n+^ layers are built up by Cd(1)O_4_F_2_N pentagonal bipyramid sharing two vertexes giving rise to chains parallel to [010] direction, liked by Cd(2)O_4_N_2_ octahedra, which share vertex with two Cd(1) from different chains. In the holes generated, there are oxalate ligands, which are linking six cadmium atoms (Figure 2).

The topological simplification of the net was made using ToposPro software [29]. The Ina and oxalate ligands were reduced to an unique atom, representing the center of the molecule (Figure S3). The structure can be described as 3,3,6,6,6-c net with stoichiometry (3-c)2(3-c)2(6-c)(6-c)2(6-c); 5-nodal net with point (Schlafli) symbol {4.8^2^}2{4^3^}2{4^6^.6^4^.8^5^}2{4^6^.6^6^.8^3^}{4^8^.6^6^.8}. This type of net was not previously described.

**Figure 3:**
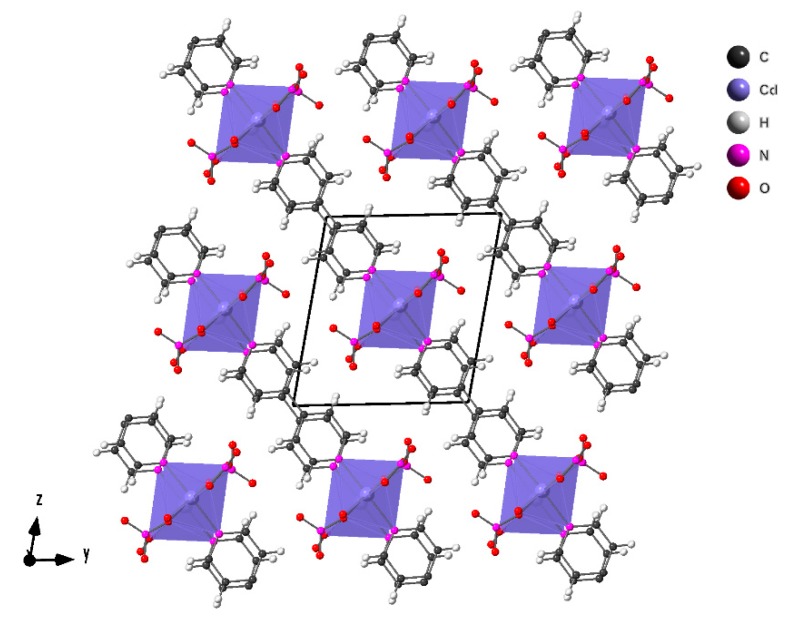
Crystal structure representation of compound **2**.

Compound **2**, with chemical formula Cd(NO_3_)_2_(4,4′-Bpy) (4,4′-Bpy = 4,4′-Bipyridine), crystallizes in the triclinic space group P*-1*. The compound has a two-dimensional framework with layers pillared perpendicular to [011] direction (Figure 3).

**Figure 4:**
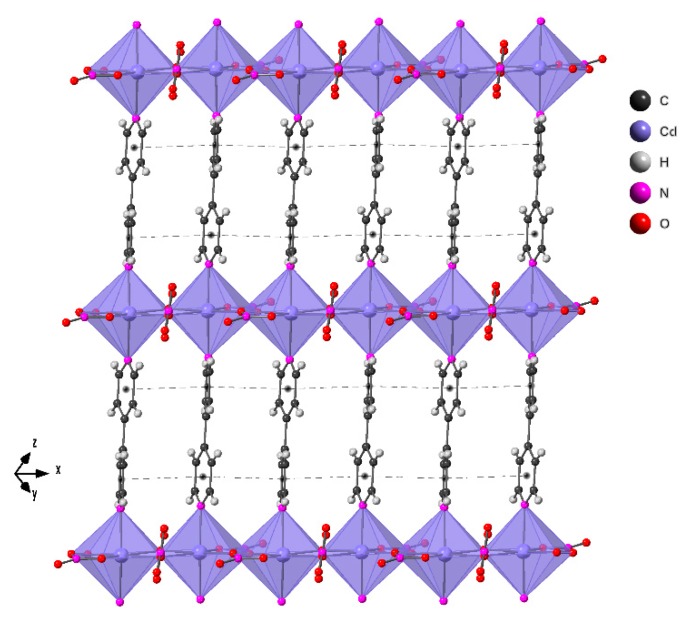
View of the layers of compound **2**. The black point into the pyridine rings represents the ring centroid and the dotted lines the π–π interactions stablished between adjacent aromatic rings.

The layers are built up from [Cd(NO_3_)_2_]_n_ chains linked by 4,4′-Bpy ligands (Figure 4). The cadmium atoms are in a CdO_5_N_2_ slightly distorted pentagonal bipyramid coordination environment. By Continuous Shape Measure of this polyhedra a value S(PBP) = 2.013 was obtained, indicating that the coordination environment of the cadmium atom is slightly distorted from the ideal pentagonal bypiramid [25]. Each cadmium atom is sharing the four equatorial μ_2_-oxygen atoms from the nitrate groups with the two adjacent cadmium atoms, giving rise to chains of vertex sharing octahedra. The fifth oxygen atom of the equatorial plane is only coordinated with one cadmium atom. The bond longest Cd–O distance is 2.500 Å and the shortest 2.328 Å. The Cd–N distances are 2.256 and 2.265 Å. The bipyridine ligands have a dihedral angle between their rings of 16˚ and the distance between the ring centroids is 3.973 Å and 3.906 Å. This geometry gives rise to intra-layer π–π interactions.

Each cadmium atom is connected to other two through two oxygen atoms of the nitrate groups and to other two cadmium centers through 4,4′-Bpy ligands. This arrangement generates a 2-D 4-connected “*sql*” net, with 4^4^ · 6^2^ vertex symbol (Figure S4) [29].

A search of the structures containing only cadmium as metal and 4,4′-Bpy as bridging ligand at the Cambridge Structural Database (CSD) has yielded 485 structures. From them, the structures that contains 4,4′-Bpy as unique ligand are 47. A study of the dimensionality of these structures reveals that the most common dimensionalities are 1D and two-dimensional (2D) (18 structures of each). The compounds with three-dimensional (3D) structure are 8 and the 0D, 3. Among the structures with 2D dimensionality the formation of square-grid networks of [Cd(4,4′-Bpy)_2_]_n_ is commonly observed (13 from 18 structures). These square-grid layers are cationic and between the layers counterions and other molecules are located [30,31,32,33,34,35,36,37].

In addition to these square-grid networks, there are 3 structures [38] very related to **2**. These compounds, with general formula [Cd(μ-X)_2_(μ-4,4′-Bpy)]_n_, where X = Cl, Br and I, crystalizes in orthorhombic space groups (P*ban* when X = Cl and Br and C*mmm* when X is I). The layers of these compounds are formed by [Cd(μ-X)_2_]_n_ chains, similar to the [Cd(NO_3_)_2_]_n_ chains of **2**. As in **2**, 4,4′-Bpy ligands are bridging cadmium atoms from contiguous chains, giving rise to 2D networks. The Cd-Cd distance in the structures reported by Hu and Englert [38] increase when increasing the halide size, from 3.775 Å for chloride to 4.142 Å for iodide. In the structure of **2** there two Cd-Cd distances, one is 4.140(2) Å and the other 3.771(2) Å, which are similar to those observed in the [Cd(μ-X)_2_(μ-4,4′-Bpy)]_n_ compounds.

### 2.3. Thermal Characterization

Thermal characterization of compound **2** was carried out by thermogravimetric analysis and by temperature-dependent X-ray diffractometry. The characterization of compound **1** has not been performed because it cannot be obtained purely.

**Figure 5:**
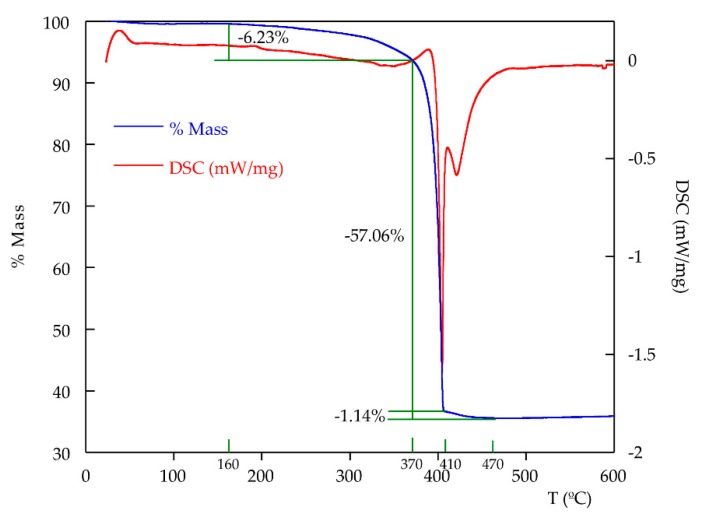
Themogravimetric curves obtained for compound **2**.

Thermogravimetric analysis of **2** shows different mass loss overlapped processes corresponding to the removal of nitrate groups and 4,4′-Bipyridine ligands (Figure 5). From 160 °C, there is a soft mass loss, which becomes sharper until 370 °C. At this point, an abrupt decrease takes place until 410 °C, followed by another soft mass decrease until 470 °C. These three processes are exothermic, as can be seen by the negative peaks in the DSC curve. The total mass loss is 64.5% (Theor.: 67.3%). The residue, analyzed by powder X-ray diffraction, is composed totally by CdO (Monteponite, S.G: F m-3m, a = 4.689 Å) [39].

**Figure 6:**
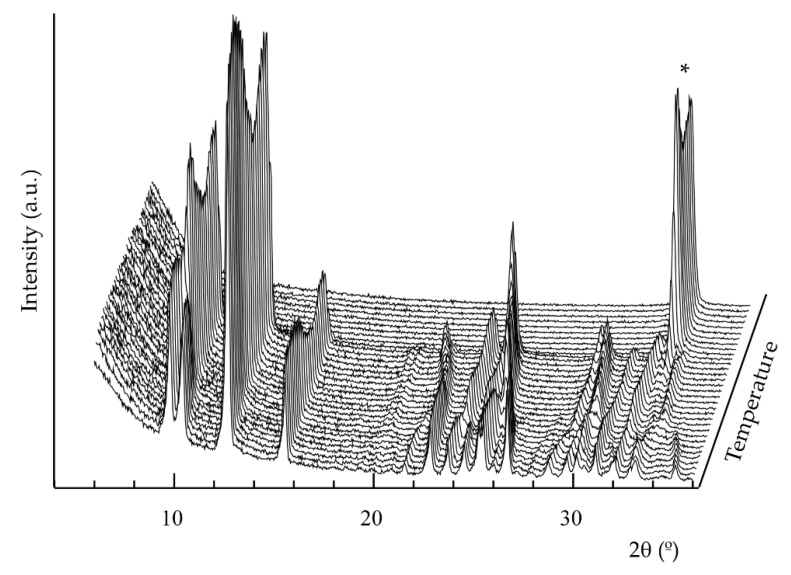
Temperature dependent X-ray diffraction analysis of **2**. The temperature variation between each diffractogram is 15 °C. * (111) peak of CdO.

On the other hand, temperature-dependent X-ray diffraction analysis was also performed (Figure 6 and Figure S5). The diffraction peaks of **2** change in position and intensity during the heating. At approximately 315 °C, the peak corresponding to the CdO residue appears, while the peaks of the phase are maintained until 360 °C. A cyclic refinement of the cell parameters from 30 to 360 °C shows that the *a* and *b* parameters increase with increasing temperature, while *c* parameter decreases. The angles α, β and γ decrease when increasing temperature (Figure S6, Table S1). The increase of *a* parameter indicates a slight elongation of the [Cd(NO_3_)_2_]_n_ chains, parallel to [100] direction, and therefore, a slight increase of Cd-Cd distances, as well as of the bypiridine ring centroids. The structural changes that take place in the structure are very progressive and do not affect to the atomic connectivity. The changes observed are due to intra-layer bond distance and angle variations.

To confirm the reversibility of the structural variations, a powdered sample was heated up to 330 °C at a heating rate of 5 °C/min and allowed to cool down again at room temperature. The diffraction pattern obtained after the heating and cooling corresponds to the mixture of **2** and CdO (Figure S7). As the conditions in the oven and in the diffractometer are not exactly the same, the formation of some CdO takes place in the oven at a lower temperature than that observed in the thermodiffractometric analysis. However, the part of the sample that did not transform to CdO gives rise to the same X-ray diffraction pattern than the pristine sample when cooled down to room temperature. This result confirms that the structural changes observed when increasing the temperature until 330 °C are due to reversible distance and angle changes.

## 3. Materials and Methods

All starting materials and solvents were obtained from reliable commercial sources and used without further purification. Ionic liquid 1-Butyl-3-methylimidazolium tetrafluoroborate ([Bmim][BF_4_]), Cd(NO_3_)_2_·4H_2_O, 4-methylpyridyne and 4,4′-Bipyridine were purchased from Sigma-Aldrich (St. Louis, MO, USA).

### 3.1. Synthesis of Cd_3_(Ox)F_2_(Ina)_2_ (1)

In a typical synthesis, a mixture of Cd(NO_3_)_2_·H_2_O (1.5 mmol, 0.463 g), 4-methylpyridyne (0.4 mmol, 38.8 μL), and 1.0 g (4.4 mmol) of [Bmim][BF_4_] was placed in a 12 mL Teflon-lined autoclave and heated up to 160 °C for 6 days. After the reaction, the autoclave was allowed to cool down to room temperature and the obtained solid was thoroughly washed with ethanol. The resulting product consisted of yellow single-crystals of **1** with tabular habit, as well as, a yellow powder, later identified as CdF_2_. A single-crystal was selected for the X-ray diffraction experiment. *Anal.* Calc for C_14_H_8_Cd_3_F_2_N_2_O_8_ (M_r_ = 707.45): C, 23.77; H, 1.14; Cd, 47.67; N, 3.96. Found: C, 23.8(1); H, 1.13(3); Cd, 47.6(2); N, 3.9(1)%.

Several tests were carried out to obtain **1** as a pure phase. The increase of the synthesis temperature favors the reduction of CdF_2_ yield. However, it does not completely disappear. Neither do other non-identified impurities. The best results were obtained when carrying out the above described procedure at 190 °C. (Figure S1(b)) This way, a beige powder is obtained and the amount of CdF_2_ is reduced. After washing the powder with water for 5 times all the CdF_2_ was disappeared, however, other unidentified impurities remain (Figure S1(c)).

### 3.2. Synthesis of Cd(4,4′-Bpy)(NO_3_)_2_ (**2**)

In the synthesis of **2** another ligand was introduced into the system, 4,4′-Bipyridine (4,4′-Bpy). In the same reaction mixture described for the synthesis of **1**, 1 mmol of 4,4′-Bpy was added (0.156 mg) and the reactor was heated up to 160 °C. After 6 days, the autoclave was allowed to cool down and the obtained product was washed with ethanol. The reaction yielded a mixture of plate light yellow crystals and yellow powder, also identified as CdF_2_. A single-crystal of **2** was selected to determine its crystal structure by X-ray diffraction. When the reaction takes place without 4-methylpyridines the same results are observed. *Anal.* Calc for C_10_H_8_CdN_4_O_6_ (M_r_ = 392.60): C, 30.59; H, 2.05; Cd, 28.63; N, 14.27. Found: C, 30.5(1); H, 2.03(3); Cd, 28.6(1); N, 14.2(1)%.

The reaction conditions were modified to obtain pure **2** varying the amount of reactants, as well as the synthesis temperature and time. The compound was obtained pure as powder when using 1.5 mmol of Cd(NO_3_)_2_·H_2_O, 0.4 mmol of 4-methylpyridine, 2 mmol of 4,4′-Bpy and 1.0 g (4.4 mmol) of [Bmim][BF_4_], and heating the mixture into a Teflon-lined autoclave at 130 °C for 6 days (Figure S2). The same results were obtained using the same conditions and reactants, but without 4-methylpyridine.

### 3.3. Single-crystal X-ray Diffraction Characterization

The crystals of **1** and **2** were selected under a polarizing microscope and mounted on MiTeGen MicroMounts™. Diffraction data were collected on Agilent Supernova single source diffractometers. Single crystal of **1** was measured with Mo Kα radiation at 100 K and crystals of **2**, with Cu Kα radiation at 150 K. Details of crystal data, intensity collection, and some features of the structural refinement are reported in Table 1. Firstly, a standard short program was used to obtain the crystal lattice and to confirm the quality of the crystals from a few diffraction images. Then, the data collection of the complete Ewald sphere was done. The diffraction data were corrected for Lorentz and polarization effects [40], as well as for the absorption, taking into account the crystal shape and size. The structure was solved by direct methods (SHELXS-2013 [41] and SHELXT [42]) and refined by the full-matrix least-squares procedure based on *F*^2^, using the SHELXL-2014 [43] computer program, included in the WINGX software package [44]. The scattering factors were taken from the International Tables for Crystallography [45]. In the case of **1**, anisotropic thermal parameters were assigned to all non H atoms. H atoms were located at Fourier maps and refined fixing their isotropic thermal parameter, assuming that the isotropic *U* value is 1.20 times the *U_eq_* value of the atom, which the hydrogen binds.

In the case of **2**, the diffraction data reveal that the crystals grow as twins. Several crystals were tested, and in all cases, the twins composed of two or more components were observed. The data reduction was made for two components of the twin with a rotation angle of 180° around the [0 0.71 -0.71] direction (Figure S8). Firstly, the intensity of the first component’s maxima were extracted and HKLF4 files were generated. Secondly, the extraction of the maxima of both components that were completely separated or completely coincident was done, generating the HKLF5 files. The structure was solved by direct methods (SHELXT [42]) taking into account the reflections of the first twin component and then the refinement was carried out by full-matrix least-squares procedure, based on *F*^2^ using the SHELXL-2014 [43] program using the reflections of both components and introducing the BASF order in the refinement. After the refinement a BASF parameter of 0.493 is obtained. Anisotropic thermal parameters were assigned to all non H atoms. The positions of the H atoms were calculated at their ideal positions and refined fixing their isotropic thermal parameter, assuming that the isotropic *U* value is 1.20 times the *U_eq_* value of the atom which the hydrogen binds.

Atomic coordinates and selected bond distances and angles are shown in Tables S2 to S7 in Supporting Information. Further details on the crystal structures can be obtained from the CCDC by quoting the depository numbers 1955019 (**1**) and 1955020 (**2**).

### 3.4. Physico-Chemical Characterization Methods

The materials were characterized by powder X-ray diffraction (XRD), elemental analysis, and inductively coupled plasma atomic emission spectroscopy (ICP-AES). XRD data were recorded on a Philips X’Pert diffractometer with secondary monochromator in the 5–70° 2θ range, with a stepsize of 0.026°.

Elemental analyses were performed on an EuroVector Euro EA Elemental Analyzer (CHNS) and ICP-AES analysis on a Horiba Yobin Yvon Activa spectrometer.

Thermogravimetric analyses were carried out in order to determine the ionic liquid loading and the water content of the MOF under synthetic air atmosphere with a NETZSCH STA 449F3 DSC–TGA instrument. A crucible containing the sample (ca. 10 mg) was heated at 5 °Cmin^–1^ in the temperature range 30–600 °C. The gas flow was 60 mL/min for the whole measure. Temperature dependent X-ray diffraction analysis was performed on a Bruker D8 Advance Vantec diffractometer equipped with an Anton Parr HTK2000 variable temperature stage with a Pt sample holder. The diffraction patterns were collected in 2θ steps of 0.0333° in the range of 6–36°, counting for 0.1 s per step and increasing the temperature at 15 °C between each diffractograms at 10 °Cmin^−1^ from 30 up to 510 °C.

## 4. Conclusions

Ionothermal synthesis has been demonstrated as an adequate method for the synthesis of coordination polymers based on cadmium(II), obtaining single-crystals using little amounts of ionic liquid solvents. The synthetic parameter such as temperature and reactants molar ratio are key in improving the purity of the product. The role of the ionic liquid in the synthesis may be more than the solvent, being in case of **1** synthesis a promoter of the ligand oxidation and the fluorine source. These reactions take place in-situ during the reaction of the final product and seem to be favored by the increase of the reaction temperature. Therefore, it is possible to obtain new compounds by ionothermal synthesis, that cannot otherwise be obtained by other synthetic methods.

## Figures and Tables

**Table 1:** Crystal data, data collection and structure refinement details for **1** and **2**.

	1	2
**Crystal data**		
Chemical formula	C_14_H_8_Cd_3_F_2_N_2_O_8_	C_10_H_8_CdN_4_O_6_
*M*_r_	707.42	392.6
Crystal system, space group	Monoclinic, *P*2_1_/*n*	Triclinic, P-1
*a*, *b*, *c* (Å)	10.4405 (4),7.6000(2), 12.2919(5)	7.842(1), 9.314(1), 10.183(2)
α, β, γ (°)	90, 113.019 (5), 90	72.72(1), 68.97(1), 69.54(1)
*V* (Å^3^)	897.67 (6)	637.8 (2)
*Z*	2	2
*D*_x_ (g cm^−3^)	2.617	2.047
*F*(000)	664	384
μ (mm^−1^)	3.59	14.10
Crystal size (mm)	0.33 × 0.10 × 0.05	0.18 × 0.07 × 0.06
**Data collection**		
Radiation type (λ)	Mo Kα (0.71073 Å)	Cu Kα (1.54184 Å)
Temperature (K)	100	150
θ range (˚)	3.2–27.2	4.7–74.3
h, k, l ranges	−13 ≤ h ≤ 12, −9 ≤ k ≤ 9, −15 ≤ l ≤ 15	−9 ≤ h ≤ 9, −11 ≤ k ≤ 10, −12 ≤ l ≤ 12
*T*_min_, *T*_max_	0.542, 0.854	0.397, 1
No. of meas. refl. (*R*_int_)	5712 (0.052)	2547 (0.057)
No. of independent and observed [*I* > 2σ(*I*)] refl.	1846, 1546	2547, 2200
(sin θ/λ)_max_ (Å^−1^)	0.643	0.626
**Refinement**		
*R*[*F*^2^ > 2σ(*F*^2^)], *wR*(*F*^2^), *S*	0.034, 0.062, 1.05	0.091, 0.265, 1.12
No. of reflections	1846	2547
No. of parameters	145	191
Largest diff. peak and hole (e Å^−3^)	0.76 and -0.82	4.80and -2.12
BASF	-	0.493

## Supplementary Materials

The following are available online at https://www.mdpi.com/1420-3049/24/22/4059/s1, Figure S1: Powder X-ray diffraction pattern-matching fittings for the samples of **1** synthesized (a) at 160 °C, (b) at 190 °C, and (c) at 190 °C and washed with water; Figure S2: Powder X-ray diffraction pattern-matching fittings for the samples of **2** synthesized at (a) 160° and (b) 130 °C; Figure S3: Topological simplification of compound **1**; (a) 3D view and (b) view of the [Cd_3_(ox)F_2_]_n_^2n+^ layers; Figure S4: Topological simplification of compound **2**; Figure S5: 2D view of the thermo-diffraction analysis of **2**; Figure S6: Evolution of the cell parameter with the temperature for **2**; Figure S7: Comparison of diffractograms of **2**, CdO and **2** heated up to 330 °C; Figure S8: Representation of the crystal cells of the two components of the twin used for the X-ray diffraction data collection of **2**; Table S1 and S4: Fractional atomic coordinates and isotropic or equivalent isotropic displacement parameters (Å2) for **1** and **2**; Table S2 and S5: Atomic displacement parameters (Å2) for **1** and **2**; Table S3 and S6: Geometric parameters (Å, °) for **1** and **2**.
