# Supplementary material for: Ionothermal Synthesis of Cadmium Coordination Polymers: Ionic Liquid Effects on the Synthesis, Structural, and Thermal Characterization
Source: Molecules. 2019 Nov 9;24(22):4059. doi: 10.3390/molecules24224059 (PMC6891532; doi:10.3390/molecules24224059)

checkCIF/PLATON report

Structure factors have been supplied for datablock(s) shelx

THIS REPORT IS FOR GUIDANCE ONLY. IF USED AS PART OF A REVIEW PROCEDURE FOR PUBLICATION, IT SHOULD NOT REPLACE THE EXPERTISE OF AN EXPERIENCED CRYSTALLOGRAPHIC REFEREE.

No syntax errors found. CIF dictionary Interpreting this report

Datablock: shelx

```
Bond precision:  C-C = 0.0087 A          Wavelength=0.71073

Cell:           a=10.4405(4)    b=7.6000(2)         c=12.2919(5)
                alpha=90        beta=113.019(5)     gamma=90
Temperature:    100 K

                Calculated                  Reported
Volume          897.68(6)                   897.67(6)
Space group     P 21/n                      P 21/n
Hall group      -P 2yn                      -P 2yn
Moiety formula  C14 H8 Cd3 F2 N2 O8         ?
Sum formula     C14 H8 Cd3 F2 N2 O8         C14 H8 Cd3 F2 N2 O8
Mr              707.45                      707.42
Dx,g cm-3       2.617                       2.617
Z               2                           2
Mu (mm-1)       3.589                       3.589
F000            664.0                       664.0
F000'           659.49
h,k,lmax        13,9,15                     13,9,15
Nref            2008                        1846
Tmin,Tmax       0.648,0.833                 0.542,0.854
Tmin'           0.302

Correction method= # Reported T Limits: Tmin=0.542 Tmax=0.854
AbsCorr = GAUSSIAN

Data completeness= 0.919          Theta(max)= 27.208

R(reflections)= 0.0339( 1546)     wR2(reflections)= 0.0621( 1846)

S = 1.049                   Npar= 145
```

The following ALERTS were generated. Each ALERT has the format
test-name_ALERT_alert-type_alert-level.
Click on the hyperlinks for more details of the test.

Alert level C

```
PLAT213_ALERT_2_C Atom O1              has ADP max/min Ratio .....        3.1 prolat
PLAT213_ALERT_2_C Atom C6              has ADP max/min Ratio .....        3.3 prolat
PLAT230_ALERT_2_C Hirshfeld Test Diff for    O2       --C6       .        5.2 s.u.
PLAT342_ALERT_3_C Low Bond Precision on  C-C Bonds ...............    0.00867 Ang.
PLAT975_ALERT_2_C Check Calcd Resid. Dens.  0.87A   From O2              0.71 eA-3
PLAT976_ALERT_2_C Check Calcd Resid. Dens.  1.03A   From O2             -0.67 eA-3
PLAT978_ALERT_2_C Number C-C Bonds with Positive Residual Density.          0 Info
```

Alert level G

```
PLAT004_ALERT_5_G Polymeric Structure Found with Maximum Dimension          3 Info
PLAT164_ALERT_4_G Nr. of Refined C-H H-Atoms in Heavy-Atom Struct.          4 Note
PLAT764_ALERT_4_G Overcomplete CIF Bond List Detected (Rep/Expd) .       1.16 Ratio
PLAT794_ALERT_5_G Tentative Bond Valency for Cd1       (II)      .       2.01 Info
PLAT794_ALERT_5_G Tentative Bond Valency for Cd2       (II)      .       2.22 Info
PLAT883_ALERT_1_G No Info/Value for _atom_sites_solution_primary .     Please Do !
PLAT910_ALERT_3_G Missing # of FCF Reflection(s) Below Theta(Min).          1 Note
PLAT912_ALERT_4_G Missing # of FCF Reflections Above STh/L=  0.600        162 Note
```

0 **ALERT level A** = Most likely a serious problem - resolve or explain
0 **ALERT level B** = A potentially serious problem, consider carefully
7 **ALERT level C** = Check. Ensure it is not caused by an omission or oversight
8 **ALERT level G** = General information/check it is not something unexpected

1 ALERT type 1 CIF construction/syntax error, inconsistent or missing data
6 ALERT type 2 Indicator that the structure model may be wrong or deficient
2 ALERT type 3 Indicator that the structure quality may be low
3 ALERT type 4 Improvement, methodology, query or suggestion
3 ALERT type 5 Informative message, check

It is advisable to attempt to resolve as many as possible of the alerts in all categories. Often the minor alerts point to easily fixed oversights, errors and omissions in your CIF or refinement strategy, so attention to these fine details can be worthwhile. In order to resolve some of the more serious problems it may be necessary to carry out additional measurements or structure refinements. However, the purpose of your study may justify the reported deviations and the more serious of these should normally be commented upon in the discussion or experimental section of a paper or in the "special_details" fields of the CIF. checkCIF was carefully designed to identify outliers and unusual parameters, but every test has its limitations and alerts that are not important in a particular case may appear. Conversely, the absence of alerts does not guarantee there are no aspects of the results needing attention. It is up to the individual to critically assess their own results and, if necessary, seek expert advice.

Publication of your CIF in IUCr journals

A basic structural check has been run on your CIF. These basic checks will be run on all CIFs submitted for publication in IUCr journals (*Acta Crystallographica*, *Journal of Applied Crystallography*, *Journal of Synchrotron Radiation*); however, if you intend to submit to *Acta Crystallographica Section C* or *E* or *IUCrData*, you should make sure that full publication checks are run on the final version of your CIF prior to submission.

Publication of your CIF in other journals

Please refer to the *Notes for Authors* of the relevant journal for any special instructions relating to CIF submission.

PLATON version of 07/08/2019; check.def file version of 30/07/2019

Datablock shelx - ellipsoid plot

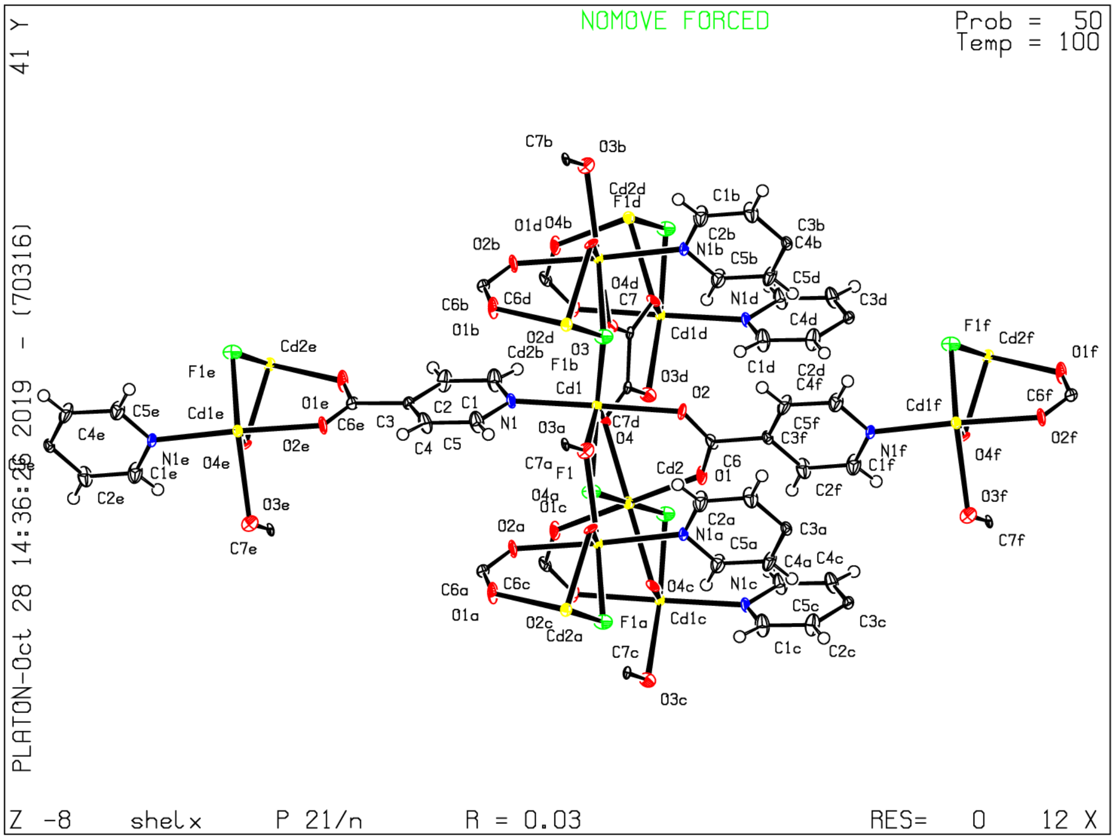

Supplement: Supplementary file 1 [file molecules-24-04059-s001.zip › checkcif_1.pdf]
