# Supplementary material for: Ionothermal Synthesis of Cadmium Coordination Polymers: Ionic Liquid Effects on the Synthesis, Structural, and Thermal Characterization
Source: Molecules. 2019 Nov 9;24(22):4059. doi: 10.3390/molecules24224059 (PMC6891532; doi:10.3390/molecules24224059)

checkCIF/PLATON report

You have not supplied any structure factors. As a result the full set of tests cannot be run.

THIS REPORT IS FOR GUIDANCE ONLY. IF USED AS PART OF A REVIEW PROCEDURE FOR PUBLICATION, IT SHOULD NOT REPLACE THE EXPERTISE OF AN EXPERIENCED CRYSTALLOGRAPHIC REFEREE.

No syntax errors found.   CIF dictionary   Interpreting this report

Datablock: shelx

```
Bond precision:  C-C = 0.0200 A          Wavelength=1.54184

Cell:          a=7.8420(9)        b=9.3142(12)      c=10.1830(16)
               alpha=72.724(13)   beta=68.977(13)   gamma=69.542(12)
Temperature:  150 K

                Calculated                Reported
Volume          637.85(16)                637.85(17)
Space group     P -1                      P -1
Hall group      -P 1                      -P 1
Moiety formula  C10 H8 Cd N4 O6           C10 H8 Cd N4 O6
Sum formula     C10 H8 Cd N4 O6           C10 H8 Cd N4 O6
Mr              392.61                    392.60
Dx,g cm-3       2.044                     2.047
Z               2                         2
Mu (mm-1)       14.085                    14.104
F000            384.0                     384.0
F000'           385.50
h,k,lmax        9,11,12                   9,11,12
Nref            2635                      2547
Tmin,Tmax       0.340,0.409               0.397,1.000
Tmin'           0.068

Correction method= # Reported T Limits: Tmin=0.397 Tmax=1.000
AbsCorr = MULTI-SCAN

Data completeness= 0.967          Theta(max)= 74.993

R(reflections)= 0.0911( 2200)     wR2(reflections)= 0.2646( 2547)

S = 1.121                 Npar= 191
```

The following ALERTS were generated. Each ALERT has the format
test-name_ALERT_alert-type_alert-level.
Click on the hyperlinks for more details of the test.

Alert level C

```
DIFMX02_ALERT_1_C  The maximum difference density is > 0.1*ZMAX*0.75
            The relevant atom site should be identified.
PLAT084_ALERT_3_C High wR2 Value (i.e. > 0.25) ...................       0.26 Report
PLAT094_ALERT_2_C Ratio of Maximum / Minimum Residual Density ....       2.26 Report
PLAT097_ALERT_2_C Large Reported Max.  (Positive) Residual Density       4.80 eA-3
PLAT213_ALERT_2_C Atom C2              has ADP max/min Ratio .....        3.1 oblate
PLAT342_ALERT_3_C Low Bond Precision on  C-C Bonds ...............       0.02 Ang.
```

Alert level G

```
PLAT004_ALERT_5_G Polymeric Structure Found with Maximum Dimension          2 Info
PLAT072_ALERT_2_G SHELXL First  Parameter in WGHT  Unusually Large       0.12 Report
PLAT083_ALERT_2_G SHELXL Second Parameter in WGHT  Unusually Large      17.18 Why ?
PLAT764_ALERT_4_G Overcomplete CIF Bond List Detected (Rep/Expd) .       1.15 Ratio
PLAT794_ALERT_5_G Tentative Bond Valency for Cd1       (II)      .       2.12 Info
PLAT870_ALERT_4_G ALERTS Related to Twinning Effects Suppressed ..          ! Info
PLAT883_ALERT_1_G No Info/Value for _atom_sites_solution_primary .     Please Do !
```

0 **ALERT level A** = Most likely a serious problem - resolve or explain
0 **ALERT level B** = A potentially serious problem, consider carefully
6 **ALERT level C** = Check. Ensure it is not caused by an omission or oversight
7 **ALERT level G** = General information/check it is not something unexpected

2 ALERT type 1 CIF construction/syntax error, inconsistent or missing data
5 ALERT type 2 Indicator that the structure model may be wrong or deficient
2 ALERT type 3 Indicator that the structure quality may be low
2 ALERT type 4 Improvement, methodology, query or suggestion
2 ALERT type 5 Informative message, check

It is advisable to attempt to resolve as many as possible of the alerts in all categories. Often the minor alerts point to easily fixed oversights, errors and omissions in your CIF or refinement strategy, so attention to these fine details can be worthwhile. In order to resolve some of the more serious problems it may be necessary to carry out additional measurements or structure refinements. However, the purpose of your study may justify the reported deviations and the more serious of these should normally be commented upon in the discussion or experimental section of a paper or in the "special_details" fields of the CIF. checkCIF was carefully designed to identify outliers and unusual parameters, but every test has its limitations and alerts that are not important in a particular case may appear. Conversely, the absence of alerts does not guarantee there are no aspects of the results needing attention. It is up to the individual to critically assess their own results and, if necessary, seek expert advice.

Publication of your CIF in IUCr journals

A basic structural check has been run on your CIF. These basic checks will be run on all CIFs submitted for publication in IUCr journals (*Acta Crystallographica*, *Journal of Applied Crystallography*, *Journal of Synchrotron Radiation*); however, if you intend to submit to *Acta Crystallographica Section C* or *E* or *IUCrData*, you should make sure that full publication checks are run on the final version of your CIF prior to submission.

Publication of your CIF in other journals

Please refer to the *Notes for Authors* of the relevant journal for any special instructions relating to CIF submission.

PLATON version of 07/08/2019; check.def file version of 30/07/2019

Datablock shelx - ellipsoid plot

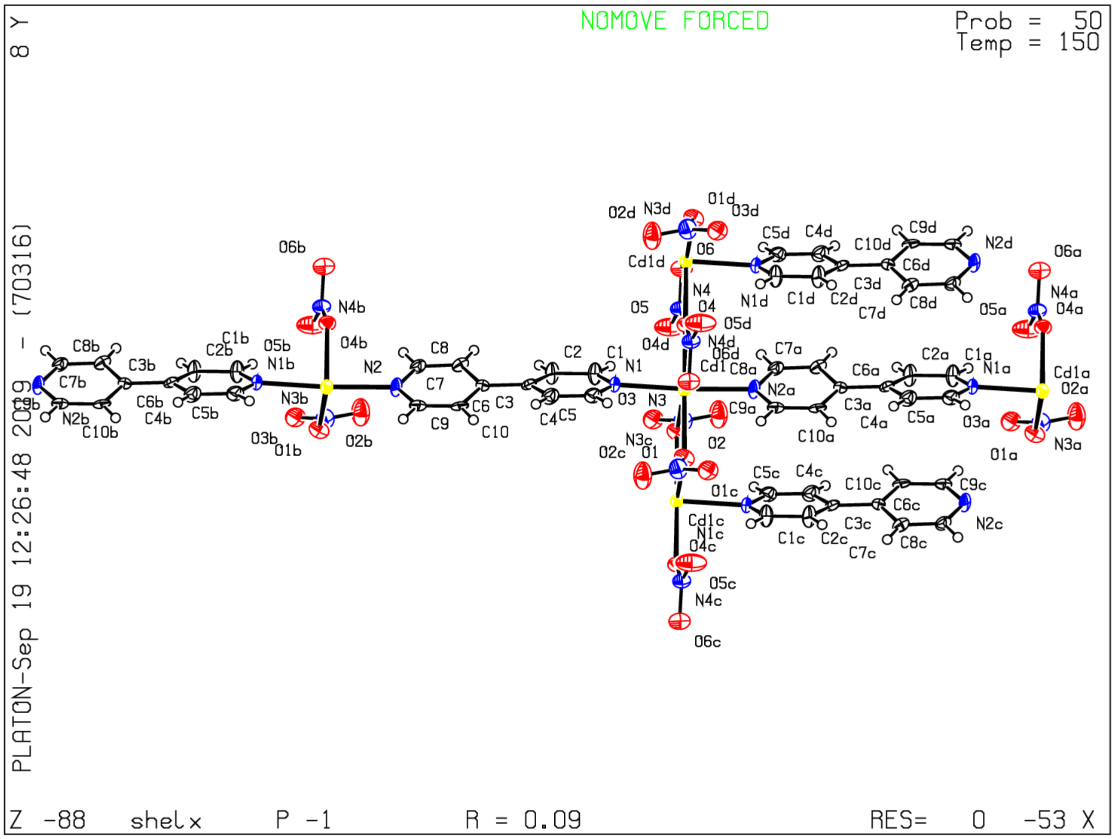

Supplement: Supplementary file 1 [file molecules-24-04059-s001.zip › checkcif_2.pdf]
