# Supplementary material for: Ionothermal Synthesis of Cadmium Coordination Polymers: Ionic Liquid Effects on the Synthesis, Structural, and Thermal Characterization
Source: Molecules. 2019 Nov 9;24(22):4059. doi: 10.3390/molecules24224059 (PMC6891532; doi:10.3390/molecules24224059)
Supplement: Supplementary file 1 [file molecules-24-04059-s001.zip › SM_623454.docx]

Ionothermal synthesis of cadmium coordination polymers: ionic liquid effects on the synthesis, structural and thermal characterization.

Iñigo PerezF ^1^, Edurne S. Larrea ^1^*, Begoña Bazán ^1,3^, Gotzone Barandika ^2,3^, M. Karmele Urtiaga ^1^, and Maria I. Arriortua ^1,3^

^1^ Dpto. Mineralogía y Petrología; ^2^ Dpto. Química Inorgánica, Universidad del País Vasco, UPV/EHU, Sarriena s/n, 48940 Leioa, Spain; inigo.perezf@ehu.eus, edurne.serrano@ehu.eus, bego.bazan@ehu.eus, gotzone.barandika@ehu.eus, karmele.urtiaga@ehu.eus, maribel.arriortua@ehu.eus

^3^ BCMaterials (Basque Center for Materials, Applications & Nanostructures), UPV/EHU Scientific Park, Martina Casiano Building, 3th floor, Sarriena s/n, Leioa, Spain

***** Correspondence: edurne.serrano@ehu.eus; Tel.: +34-946-015-984

Figure S1: Powder X-ray diffraction pattern-matching fittings for the samples of **1** synthesized (a) at 160 °C, (b) at 190 °C and (c) at 190 °C and washed with water.

Figure S.2: Powder X-ray diffraction pattern-matching fittings for the samples of **2** synthesized at (a) 160 ° and (b) 130 °C.

Figure S.3: Topological simplification of compound **1**; (a) 3D view and (b) view of the [Cd_3_(ox)F_2_]_n_^2n+^ layers.

Figure S.4: Topological simplification of compound **2**.

Figure S.5: 2D view of the thermo-diffraction analysis of **2**.

Figure S.6: Evolution of the cell parameter with the temperature for **2**.

Figure S.7: Comparison of diffractograms of **2**, CdO and **2** heated up to 330 °C.

Figure S.8: Representation of the crystal cells of the two components of the twin used for the X-ray diffraction data collection of **2**.

Table S.1: Fractional atomic coordinates and isotropic or equivalent isotropic displacement parameters (Å2) for **1**.

Table S.2: Atomic displacement parameters (Å2) for **1**.

Table S.3: Geometric parameters (Å, °) for **1**.

Table S.4: Fractional atomic coordinates and isotropic or equivalent isotropic displacement parameters (Å2) for **2**.

Table S.5: Atomic displacement parameters (Å2) for **2**.

Table S.6: Geometric parameters (Å, °) for **2**.

| 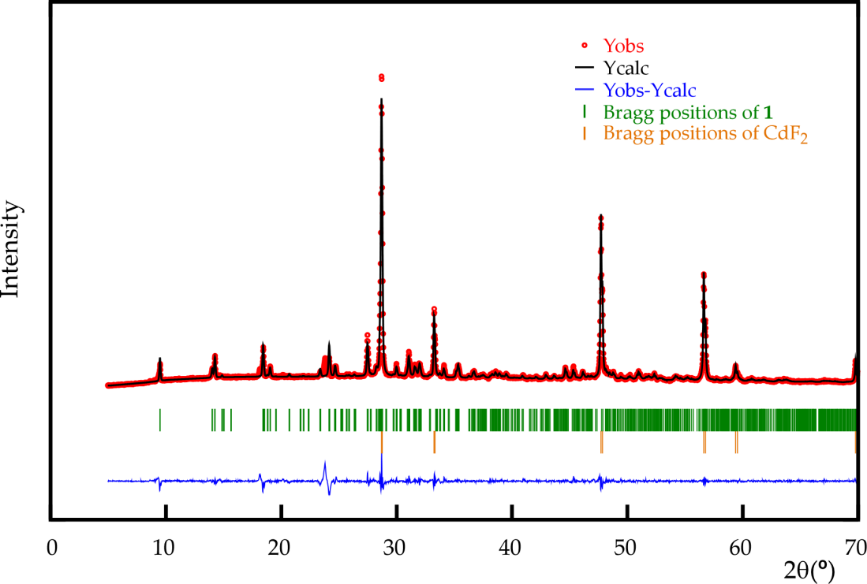 | (a) |
| --- | --- |
| 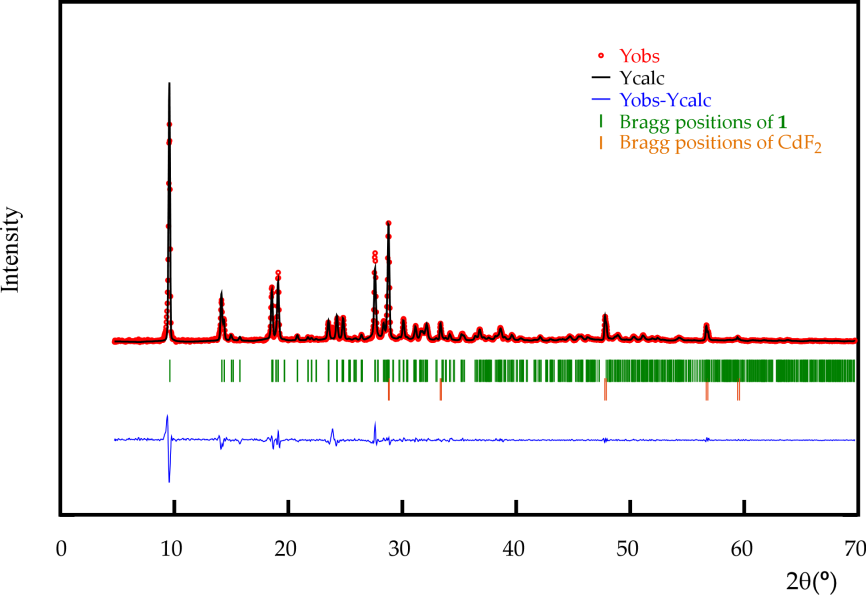 | (b) |
| 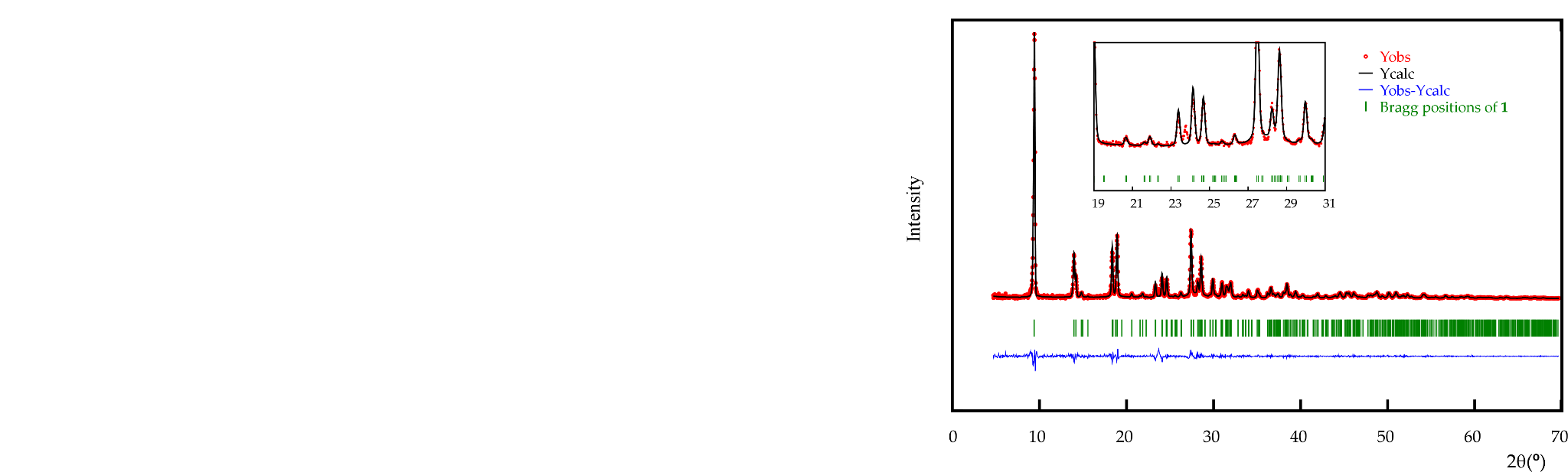 | (c) |

Figure S.1. Powder X-ray diffraction pattern-matching fittings for the samples of **1** synthesized (a) at 160 ºC, (b) at 190 ºC and (c) at 190 ºC and washed with water. In the inlet of (c) peak corresponding to a non-identified impurity is observed.

| 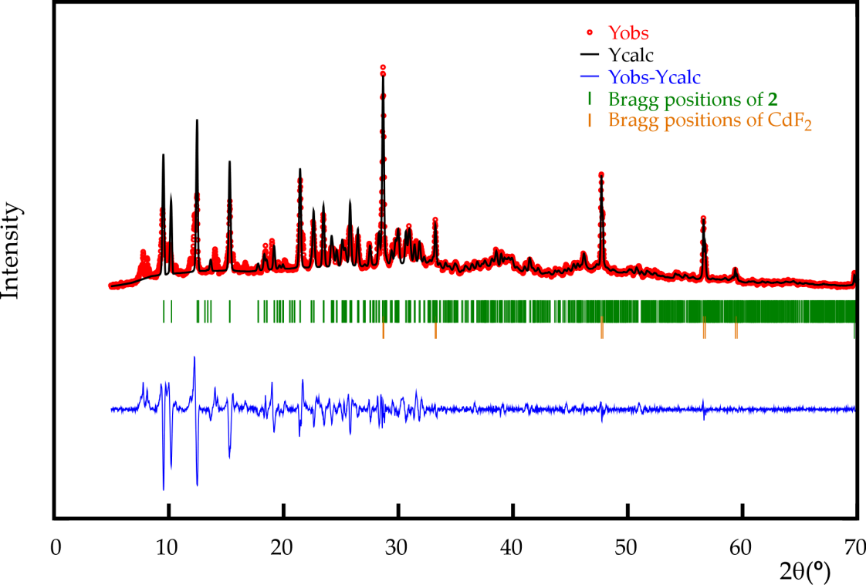 | (a) |
| --- | --- |
| 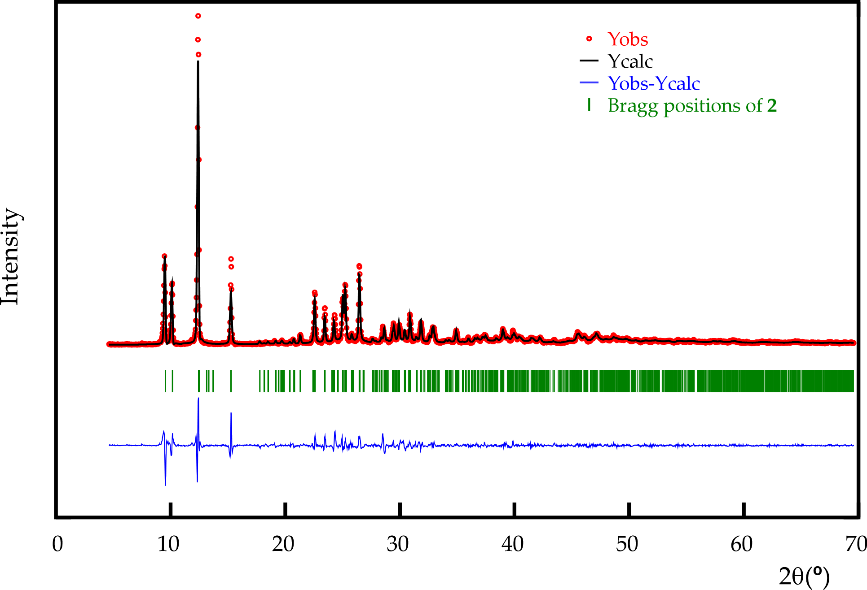 | (b) |

Figure S.2. Powder X-ray diffraction pattern-matching fittings for the samples of **2** synthesized at (a) 160 º and (b) 130 ºC.

| 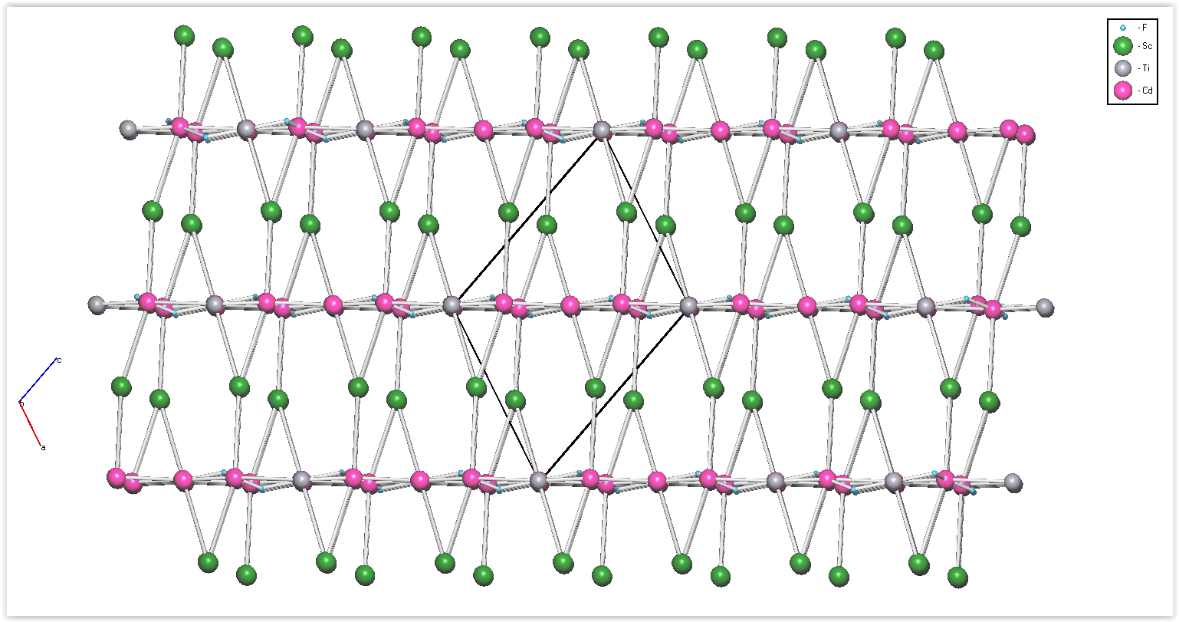 | (a) |
| --- | --- |
| 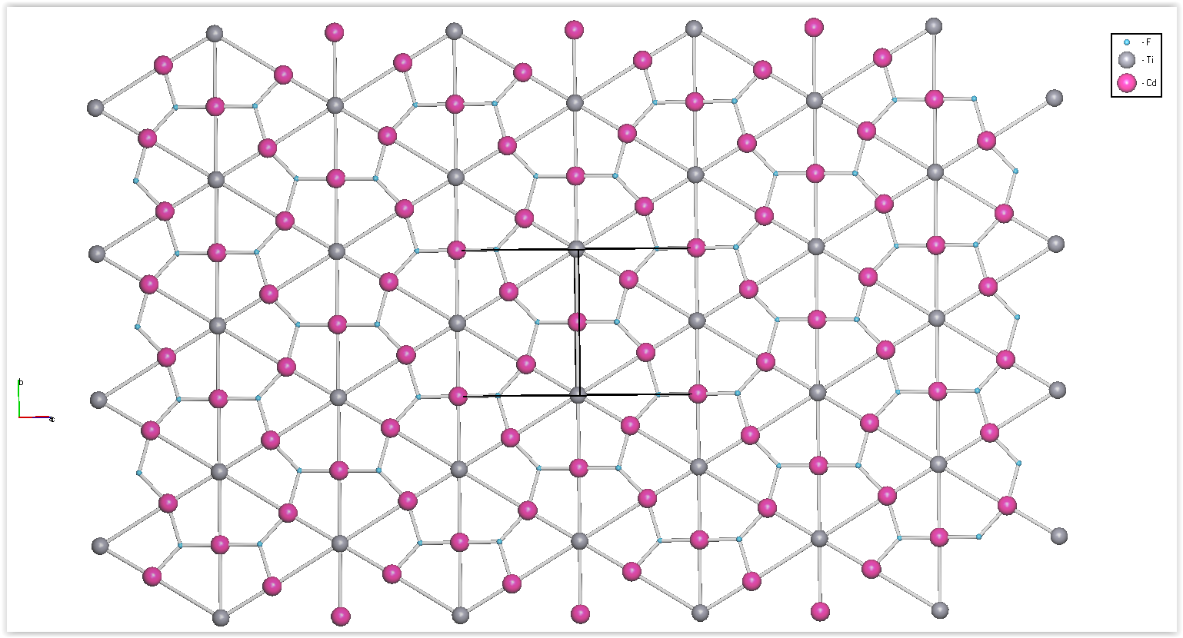 | (b) |

Figure S. 3. Topological simplification of compound **1**; (a) 3D view and (b) view of the [Cd_3_(ox)F_2_]_n_^2n+^ layers. Purple spheres corresponds to cadmium metal centers, green spheres to isonicotinate ligands, grey spheres to oxalate ligand and cyan spheres to fluoride atoms.


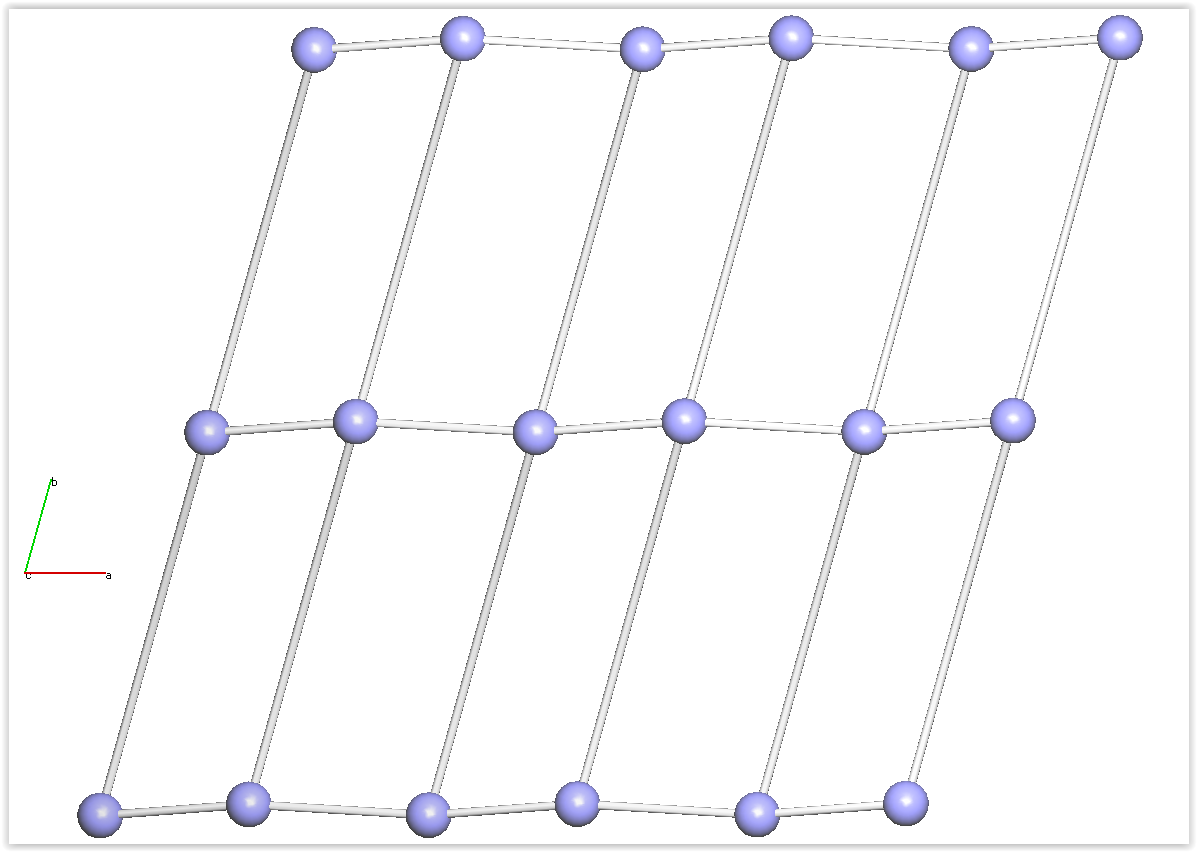


Figure S. 4. Topological simplification of compound **2**. Purple spheres corresponds to cadmium metal centers and grey bars to 4-Bpy ligands and oxo-bridges.


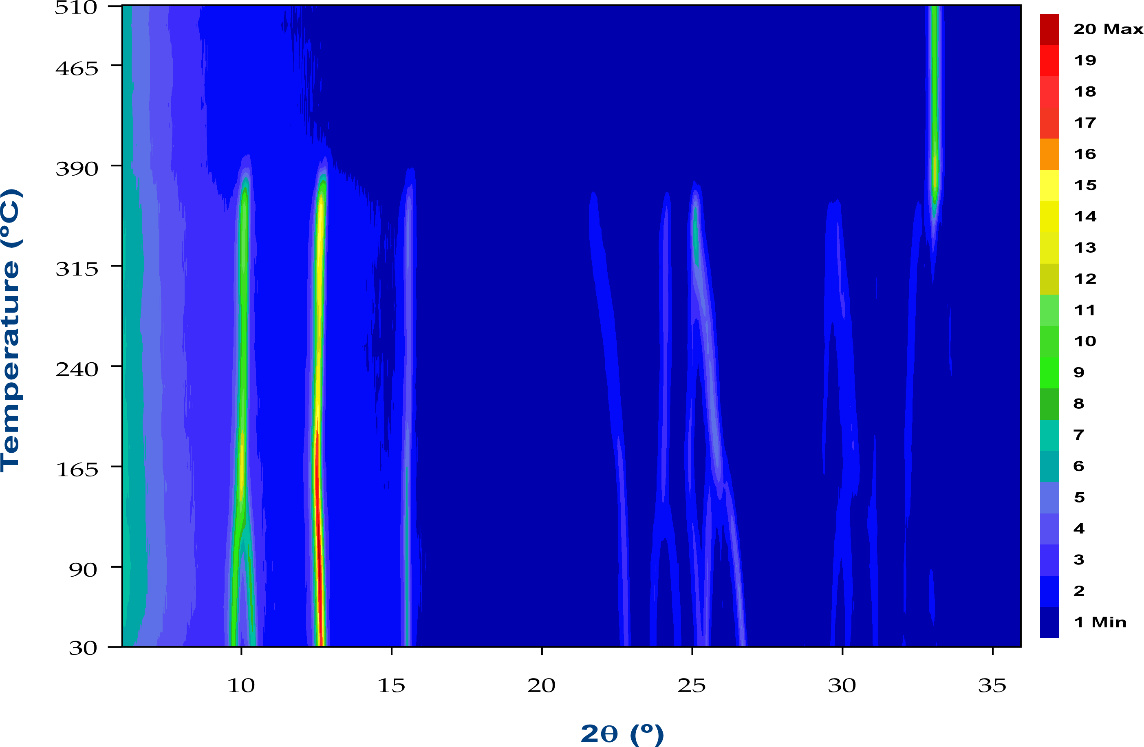


Figure S. 5. 2D view of the thermo-diffraction analysis of **2**.


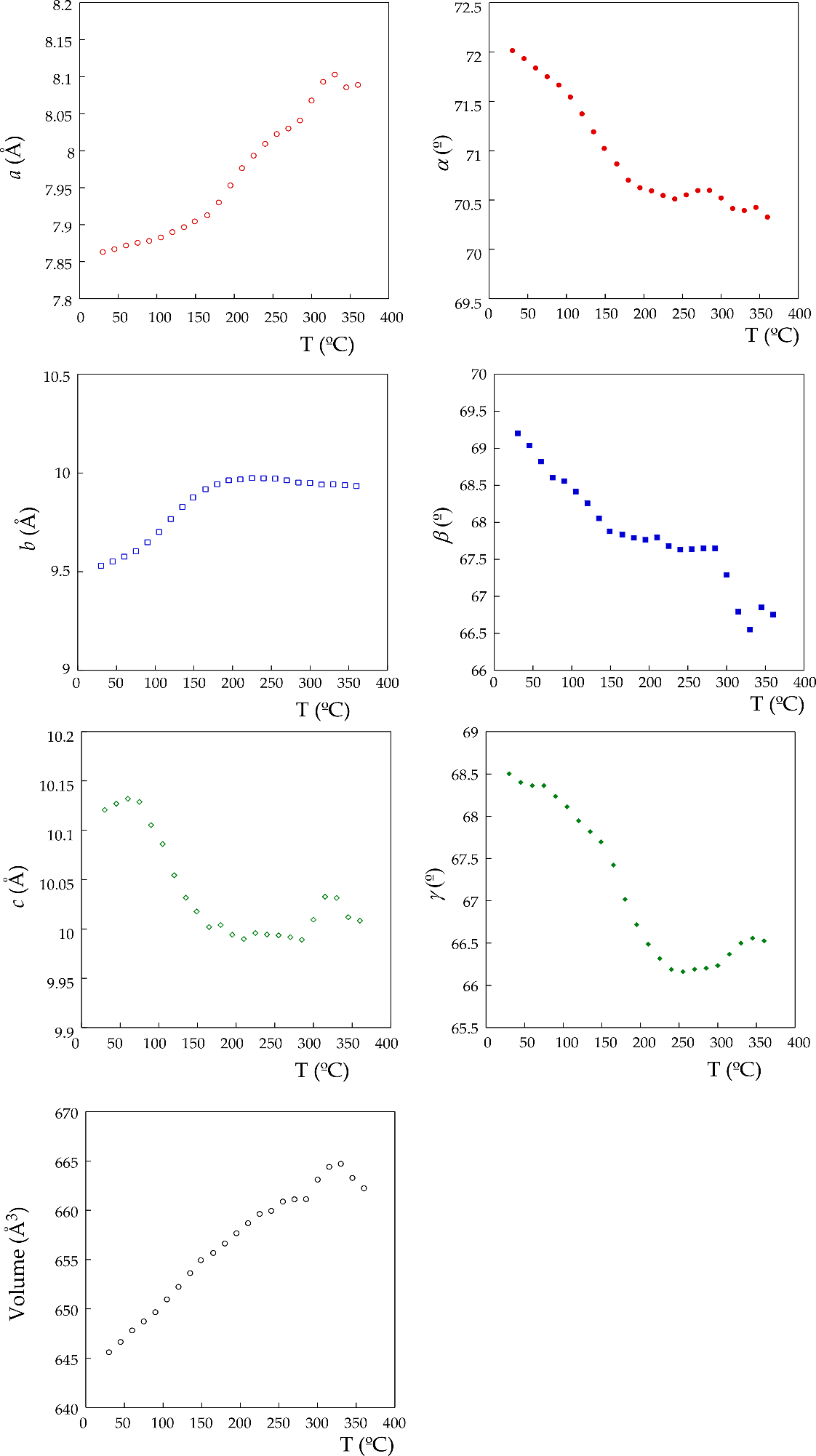


Figure S. 6. Evolution of the cell parameter with the temperature for **2**.


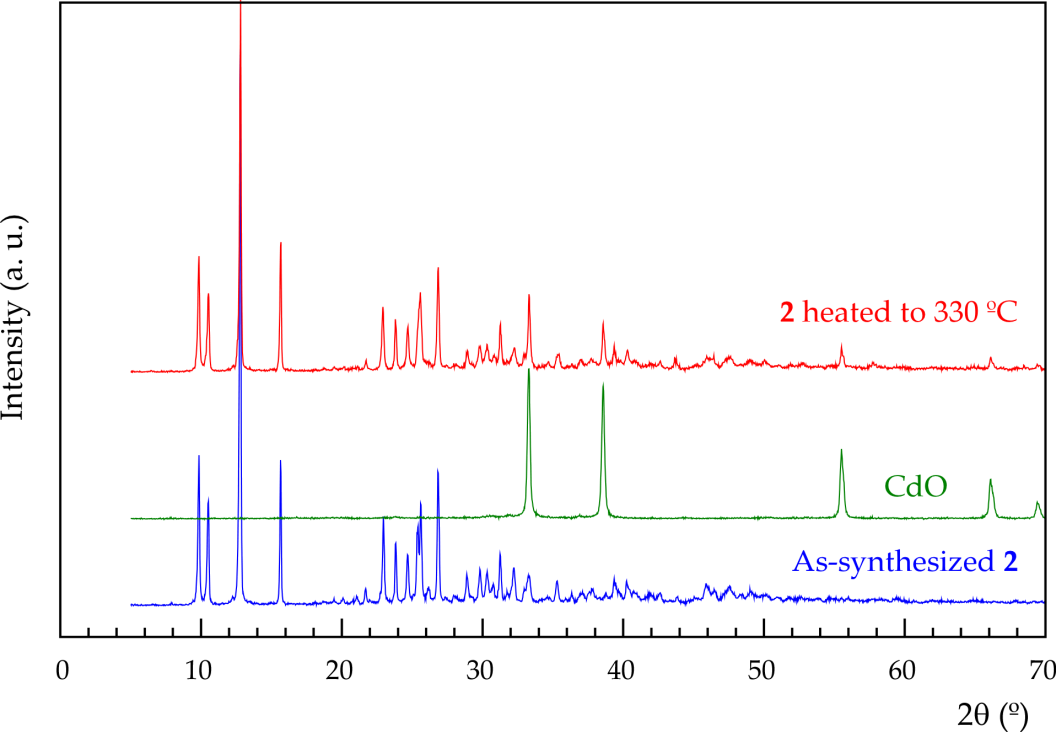


Figure S.7. Comparison of diffractograms of **2**, CdO and **2** heated up to 330 ºC.


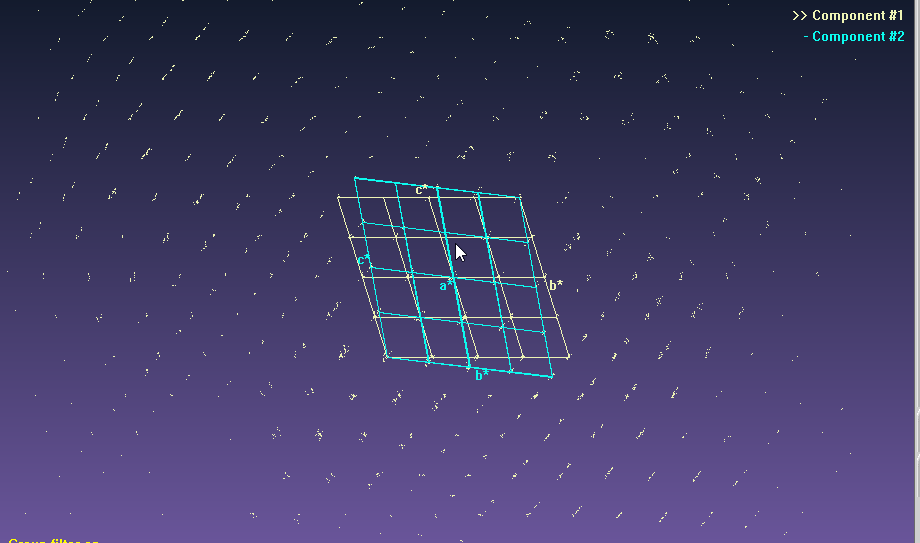


Figure S.8. Representation of the reciprocal space and the crystal cells of the two components of the twin used for the X-ray diffraction data collection of **2**. Twin law: (-1.00 -0.04 0.05 0.00 -0.12 -0.87 0 -1.13 0.12)

Table S.1. Results for the cyclic refinement of the cell parameters of **1** with the temperature.

| T (º) | *a* (Å) | *b* (Å) | *c* (Å) | α (º) | β (º) | γ (º) | V (Å^3^) | Chi^2^ |
| --- | --- | --- | --- | --- | --- | --- | --- | --- |
| 30 | 7.867(1) | 9.552(3) | 10.127(2) | 71.93(1) | 69.04(2) | 68.40(3) | 646.6(2) | 9.48113 |
| 45 | 7.871(1) | 9.576(3) | 10.132(2) | 71.84(1) | 68.82(3) | 68.36(3) | 647.8(3) | 14.03730 |
| 60 | 7.876(1) | 9.604(3) | 10.129(3) | 71.75(1) | 68.60(3) | 68.36(3) | 648.7(3) | 15.14070 |
| 75 | 7.878(1) | 9.649(3) | 10.105(3) | 71.66(1) | 68.56(3) | 68.24(3) | 649.7(3) | 14.32010 |
| 90 | 7.883(1) | 9.701(3) | 10.086(3) | 71.54(1) | 68.42(3) | 68.11(3) | 651.0(3) | 13.25300 |
| 105 | 7.890(1) | 9.767(3) | 10.054(3) | 71.37(1) | 68.26(3) | 67.94(2) | 652.2(3) | 12.31230 |
| 120 | 7.897(1) | 9.828(3) | 10.032(2) | 71.19(1) | 68.06(2) | 67.82(3) | 653.6(3) | 9.72654 |
| 135 | 7.904(2) | 9.876(3) | 10.018(2) | 71.02(2) | 67.88(2) | 67.70(3) | 654.9(3) | 10.81810 |
| 150 | 7.913(1) | 9.918(3) | 10.002(2) | 70.87(2) | 67.84(2) | 67.42(2) | 655.7(2) | 7.75308 |
| 165 | 7.930(2) | 9.943(3) | 10.004(2) | 70.70(2) | 67.79(2) | 67.02(3) | 656.6(3) | 8.14998 |
| 180 | 7.953(2) | 9.962(3) | 9.994(2) | 70.62(2) | 67.77(3) | 66.72(3) | 657.7(3) | 7.03577 |
| 195 | 7.976(2) | 9.967(3) | 9.990(3) | 70.59(2) | 67.80(3) | 66.49(3) | 658.7(3) | 8.17320 |
| 210 | 7.993(2) | 9.974(3) | 9.996(3) | 70.55(2) | 67.68(3) | 66.32(3) | 659.6(3) | 8.55598 |
| 225 | 8.009(2) | 9.973(3) | 9.994(3) | 70.51(2) | 67.63(3) | 66.19(3) | 660.0(3) | 8.28860 |
| 240 | 8.022(2) | 9.971(3) | 9.994(4) | 70.55(2) | 67.64(4) | 66.16(3) | 660.9(4) | 10.35440 |
| 255 | 8.030(2) | 9.962(2) | 9.992(3) | 70.60(2) | 67.65(3) | 66.19(2) | 661.1(3) | 8.34696 |
| 270 | 8.041(2) | 9.952(2) | 9.989(3) | 70.60(2) | 67.65(3) | 66.20(2) | 661.1(3) | 7.47688 |
| 285 | 8.068(2) | 9.950(2) | 10.010(3) | 70.52(2) | 67.29(4) | 66.24(2) | 663.1(3) | 8.16112 |
| 300 | 8.093(3) | 9.940(2) | 10.033(3) | 70.41(2) | 66.80(4) | 66.37(2) | 664.4(3) | 7.41204 |
| 315 | 8.103(5) | 9.942(2) | 10.032(4) | 70.39(2) | 66.55(5) | 66.50(2) | 664.7(5) | 7.73316 |
| 345 | 8.086(4) | 9.937(2) | 10.012(4) | 70.42(2) | 66.85(4) | 66.56(2) | 663.3(4) | 8.84372 |
| 360 | 8.089(6) | 9.934(3) | 10.008(8) | 70.33(4) | 66.76(8) | 66.52(4) | 662.2(7) | 13.56490 |

Table S.2. Fractional atomic coordinates and isotropic or equivalent isotropic displacement parameters (Å2) for **1**.

|  | x | y | z | Uiso*/Ueq |
| --- | --- | --- | --- | --- |
| Cd1 | 0.70876 (4) | 0.78726 (5) | 0.72227 (3) | 0.00827 (12) |
| Cd2 | 0.5 | 0.5 | 0.5 | 0.00986 (14) |
| O1 | 0.6769 (4) | 0.5778 (5) | 0.4507 (3) | 0.0205 (9) |
| O2 | 0.8094 (4) | 0.7671 (5) | 0.5912 (3) | 0.0171 (9) |
| O3 | 0.6218 (4) | 1.0894 (5) | 0.6317 (3) | 0.0153 (8) |
| O4 | 0.4872 (4) | 0.7872 (4) | 0.5490 (3) | 0.0123 (8) |
| F1 | 0.6418 (3) | 0.4983 (4) | 0.6880 (3) | 0.0168 (7) |
| N1 | 0.5694 (5) | 0.8119 (5) | 0.8274 (4) | 0.0132 (10) |
| C1 | 0.4523 (6) | 0.9050 (8) | 0.7845 (5) | 0.0231 (15) |
| C2 | 0.3559 (6) | 0.9105 (8) | 0.8355 (5) | 0.0203 (14) |
| C3 | 0.3831 (5) | 0.8176 (6) | 0.9399 (5) | 0.0108 (12) |
| C4 | 0.5058 (7) | 0.7256 (8) | 0.9867 (5) | 0.0219 (14) |
| C5 | 0.5954 (6) | 0.7228 (8) | 0.9276 (6) | 0.0223 (14) |
| C6 | 0.7811 (6) | 0.6754 (7) | 0.5007 (6) | 0.0173 (13) |
| C7 | 0.5378 (5) | 1.0877 (6) | 0.5240 (5) | 0.0116 (12) |
| H1 | 0.434 (6) | 0.954 (7) | 0.718 (5) | 0.028* |
| H2 | 0.274 (5) | 0.972 (7) | 0.805 (5) | 0.024* |
| H4 | 0.529 (6) | 0.675 (7) | 1.056 (5) | 0.026* |
| H5 | 0.682 (6) | 0.664 (7) | 0.961 (5) | 0.027* |

Table S.3. Atomic displacement parameters (Å2) for **1**.

|  | U11 | U22 | U33 | U12 | U13 | U23 |
| --- | --- | --- | --- | --- | --- | --- |
| Cd1 | 0.0079 (2) | 0.0093 (2) | 0.0089 (2) | -0.00106 (15) | 0.00463 (17) | -0.00132 (15) |
| Cd2 | 0.0088 (3) | 0.0114 (3) | 0.0095 (3) | -0.0028 (2) | 0.0037 (2) | -0.0015 (2) |
| O1 | 0.016 (2) | 0.029 (2) | 0.023 (2) | -0.0119 (19) | 0.016 (2) | -0.0103 (19) |
| O2 | 0.023 (2) | 0.019 (2) | 0.017 (2) | -0.0038 (18) | 0.017 (2) | -0.0066 (17) |
| O3 | 0.015 (2) | 0.016 (2) | 0.011 (2) | -0.0053 (17) | 0.0010 (18) | -0.0016 (16) |
| O4 | 0.017 (2) | 0.0074 (18) | 0.015 (2) | 0.0018 (16) | 0.0086 (18) | 0.0066 (16) |
| F1 | 0.0161 (17) | 0.0147 (16) | 0.0140 (18) | 0.0000 (14) | -0.0001 (15) | -0.0002 (13) |
| N1 | 0.016 (3) | 0.013 (2) | 0.018 (3) | 0.003 (2) | 0.014 (2) | 0.0039 (19) |
| C1 | 0.020 (3) | 0.035 (4) | 0.016 (4) | 0.008 (3) | 0.009 (3) | 0.009 (3) |
| C2 | 0.011 (3) | 0.033 (4) | 0.016 (3) | 0.009 (3) | 0.005 (3) | 0.004 (3) |
| C3 | 0.009 (3) | 0.012 (3) | 0.014 (3) | -0.002 (2) | 0.007 (3) | -0.003 (2) |
| C4 | 0.024 (4) | 0.030 (4) | 0.014 (3) | 0.001 (3) | 0.010 (3) | 0.009 (3) |
| C5 | 0.018 (3) | 0.029 (4) | 0.024 (4) | 0.010 (3) | 0.013 (3) | 0.012 (3) |
| C6 | 0.014 (3) | 0.013 (3) | 0.033 (4) | 0.005 (2) | 0.019 (3) | 0.007 (3) |
| C7 | 0.013 (3) | 0.011 (3) | 0.017 (3) | 0.001 (2) | 0.013 (3) | -0.001 (2) |

Table S.4. Geometric parameters (Å, º) for **1**.

| Cd1—F1^i^ | 2.208 (3) | O3—C7 | 1.271 (6) |
| --- | --- | --- | --- |
| Cd1—O2 | 2.247 (3) | O3—Cd1^i^ | 2.476 (4) |
| Cd1—F1 | 2.293 (3) | O4—C7^iv^ | 1.262 (6) |
| Cd1—N1 | 2.301 (4) | F1—Cd1^ii^ | 2.208 (3) |
| Cd1—O4 | 2.456 (4) | N1—C1 | 1.330 (7) |
| Cd1—O3^ii^ | 2.476 (4) | N1—C5 | 1.337 (7) |
| Cd1—O3 | 2.558 (4) | C1—C2 | 1.379 (7) |
| Cd2—F1 | 2.204 (3) | C2—C3 | 1.393 (7) |
| Cd2—F1^iii^ | 2.204 (3) | C3—C4 | 1.372 (8) |
| Cd2—O1 | 2.237 (3) | C3—C6v | 1.523 (6) |
| Cd2—O1^iii^ | 2.237 (3) | C4—C5 | 1.390 (7) |
| Cd2—O4^iii^ | 2.282 (3) | C6—C3^vi^ | 1.523 (6) |
| Cd2—O4 | 2.282 (3) | C7—O4^iv^ | 1.262 (6) |
| O1—C6 | 1.260 (6) | C7—C7^iv^ | 1.544 (10) |
| O2—C6 | 1.246 (7) |  |  |
|  |  |  |  |
| F1^i^—Cd1—O2 | 88.31 (12) | F1^iii^—Cd2—O4 | 100.74 (12) |
| F1^i^—Cd1—F1 | 153.16 (7) | O1—Cd2—O4 | 87.61 (12) |
| O2—Cd1—F1 | 89.85 (11) | O1^iii^—Cd2—O4 | 92.39 (12) |
| F1^i^—Cd1—N1 | 97.97 (13) | O4^iii^—Cd2—O4 | 180 |
| O2—Cd1—N1 | 169.84 (16) | C6—O1—Cd2 | 132.1 (3) |
| F1—Cd1—N1 | 88.11 (13) | C6—O2—Cd1 | 131.6 (3) |
| F1^i^—Cd1—O4 | 132.46 (11) | C7—O3—Cd1^i^ | 140.1 (3) |
| O2—Cd1—O4 | 85.63 (13) | C7—O3—Cd1 | 115.3 (3) |
| F1—Cd1—O4 | 74.00 (11) | Cd1^i^—O3—Cd1 | 101.43 (13) |
| N1—Cd1—O4 | 84.24 (14) | C7^iv^—O4—Cd2 | 123.4 (3) |
| F1^i^—Cd1—O3^ii^ | 85.07 (11) | C7^iv^—O4—Cd1 | 118.6 (3) |
| O2—Cd1—O3^ii^ | 94.37 (12) | Cd2—O4—Cd1 | 95.56 (12) |
| F1—Cd1—O3^ii^ | 68.38 (11) | Cd2—F1—Cd1^ii^ | 125.57 (13) |

Table S.4*. (Cont.)*

| N1—Cd1—O3^ii^ | 94.12 (13) | Cd2—F1—Cd1 | 102.62 (11) |
| --- | --- | --- | --- |
| O4—Cd1—O3^ii^ | 142.38 (12) | Cd1^ii^—F1—Cd1 | 119.91 (13) |
| F1^i^—Cd1—O3 | 68.09 (11) | C1—N1—C5 | 117.3 (4) |
| O2—Cd1—O3 | 86.29 (12) | C1—N1—Cd1 | 120.3 (4) |
| F1—Cd1—O3 | 138.48 (11) | C5—N1—Cd1 | 122.1 (4) |
| N1—Cd1—O3 | 88.64 (13) | N1—C1—C2 | 123.8 (5) |
| O4—Cd1—O3 | 64.49 (11) | C1—C2—C3 | 118.8 (5) |
| O3^ii^—Cd1—O3 | 153.14 (10) | C4—C3—C2 | 117.7 (5) |
| F1—Cd2—F1^iii^ | 180 | C4—C3—C6^v^ | 122.0 (5) |
| F1—Cd2—O1 | 89.91 (12) | C2—C3—C6^v^ | 120.3 (5) |
| F1^iii^—Cd2—O1 | 90.09 (12) | C3—C4—C5 | 119.8 (5) |
| F1—Cd2—O1^iii^ | 90.09 (12) | N1—C5—C4 | 122.5 (5) |
| F1^iii^—Cd2—O1^iii^ | 89.91 (12) | O2—C6—O1 | 128.5 (5) |
| O1—Cd2—O1^iii^ | 180 | O2—C6—C3^vi^ | 116.8 (5) |
| F1—Cd2—O4^iii^ | 100.74 (12) | O1—C6—C3^vi^ | 114.7 (5) |
| F1^iii^—Cd2—O4^iii^ | 79.26 (12) | O4^iv^—C7—O3 | 126.9 (5) |
| O1—Cd2—O4^iii^ | 92.39 (12) | O4^iv^—C7—C7^iv^ | 116.5 (6) |
| O1^iii^—Cd2—O4^iii^ | 87.61 (12) | O3—C7—C7^iv^ | 116.6 (6) |
| F1—Cd2—O4 | 79.26 (12) |  |  |

Symmetry codes: (i) -x+3/2, y+1/2, -z+3/2; (ii) -x+3/2, y-1/2, -z+3/2; (iii) -x+1, -y+1, -z+1; (iv) -x+1, -y+2, -z+1; (v) x-1/2, -y+3/2, z+1/2; (vi) x+1/2, -y+3/2, z-1/2.

Table S.5. Fractional atomic coordinates and isotropic or equivalent isotropic displacement parameters (Å2) for **2**.

|  | *x* | *y* | *z* | *U*_iso_*/*U*_eq_ |
| --- | --- | --- | --- | --- |
| Cd1 | 0.22209 (14) | 0.51524 (12) | 1.02089 (11) | 0.0216 (4) |
| N1 | 0.2406 (18) | 0.7075 (14) | 0.8225 (13) | 0.022 (3) |
| N2 | 0.226 (2) | 1.3145 (15) | 0.2107 (12) | 0.025 (3) |
| N3 | 0.219 (2) | 0.2857 (18) | 0.8326 (16) | 0.036 (3) |
| N4 | 0.6784 (18) | 0.3221 (16) | 0.8220 (15) | 0.028 (3) |
| C1 | 0.335 (3) | 0.6753 (19) | 0.6947 (17) | 0.031 (4) |
| C2 | 0.338 (3) | 0.792 (2) | 0.5716 (16) | 0.031 (4) |
| C3 | 0.242 (2) | 0.9420 (19) | 0.5796 (16) | 0.024 (3) |
| C4 | 0.139 (2) | 0.976 (2) | 0.7176 (18) | 0.030 (3) |
| C5 | 0.145 (2) | 0.854 (2) | 0.8347 (17) | 0.029 (3) |
| C6 | 0.2361 (19) | 1.0719 (17) | 0.4501 (16) | 0.021 (3) |
| C7 | 0.369 (2) | 1.0529 (19) | 0.3176 (17) | 0.025 (3) |
| C8 | 0.359 (2) | 1.1749 (19) | 0.2027 (17) | 0.025 (3) |
| C9 | 0.104 (2) | 1.333 (2) | 0.3386 (18) | 0.027 (3) |
| C10 | 0.101 (2) | 1.219 (2) | 0.4589 (18) | 0.027 (3) |
| O1 | 0.1149 (15) | 0.4037 (14) | 0.8999 (12) | 0.030 (2) |
| O2 | 0.265 (2) | 0.1563 (17) | 0.9055 (16) | 0.055 (4) |
| O3 | 0.2578 (18) | 0.3106 (17) | 0.7011 (13) | 0.040 (3) |
| O4 | 0.5580 (14) | 0.3965 (13) | 0.9143 (12) | 0.028 (2) |
| O5 | 0.6408 (19) | 0.278 (2) | 0.7411 (19) | 0.058 (4) |
| O6 | 0.8544 (16) | 0.3064 (15) | 0.8124 (13) | 0.035 (3) |
| H1 | 0.402 | 0.5725 | 0.6862 | 0.037* |
| H2 | 0.408 | 0.7653 | 0.4824 | 0.038* |
| H4 | 0.0704 | 1.0779 | 0.7293 | 0.035* |
| H5 | 0.0791 | 0.8754 | 0.9259 | 0.034* |
| H7 | 0.4626 | 0.9591 | 0.3072 | 0.031* |
| H8 | 0.4486 | 1.1613 | 0.1147 | 0.03* |
| H9 | 0.015 | 1.4298 | 0.3464 | 0.032* |
| H10 | 0.0111 | 1.2375 | 0.5457 | 0.032* |

Table S.6. Atomic displacement parameters (Å2) for **2**.

|  | *U*^11^ | *U*^22^ | *U*^33^ | *U*^12^ | *U*^13^ | *U*^23^ |
| --- | --- | --- | --- | --- | --- | --- |
| Cd1 | 0.0214 (5) | 0.0242 (6) | 0.0162 (6) | -0.0068 (4) | -0.0047 (4) | 0.0004 (4) |
| N1 | 0.025 (7) | 0.022 (6) | 0.017 (6) | -0.009 (5) | -0.007 (5) | 0.005 (5) |
| N2 | 0.030 (7) | 0.026 (6) | 0.007 (5) | -0.005 (5) | 0.003 (5) | -0.001 (5) |
| N3 | 0.035 (7) | 0.038 (8) | 0.026 (7) | -0.003 (6) | -0.002 (6) | -0.011 (6) |
| N4 | 0.024 (6) | 0.032 (7) | 0.032 (7) | -0.010 (5) | -0.008 (6) | -0.010 (6) |
| C1 | 0.049 (11) | 0.024 (8) | 0.017 (8) | -0.009 (7) | -0.008 (7) | -0.003 (6) |
| C2 | 0.044 (10) | 0.035 (9) | 0.006 (7) | -0.008 (7) | 0.001 (6) | -0.004 (6) |
| C3 | 0.017 (7) | 0.037 (8) | 0.021 (7) | -0.017 (6) | -0.004 (6) | 0.001 (6) |
| C4 | 0.024 (8) | 0.033 (8) | 0.027 (8) | -0.008 (7) | 0.000 (6) | -0.007 (7) |
| C5 | 0.020 (8) | 0.041 (9) | 0.020 (8) | -0.007 (7) | 0.000 (6) | -0.008 (7) |
| C6 | 0.013 (6) | 0.024 (7) | 0.024 (7) | -0.004 (5) | -0.006 (5) | 0.001 (6) |
| C7 | 0.021 (8) | 0.031 (8) | 0.024 (8) | -0.012 (6) | 0.000 (6) | -0.007 (6) |
| C8 | 0.017 (7) | 0.029 (8) | 0.023 (8) | -0.007 (6) | 0.002 (6) | -0.005 (6) |
| C9 | 0.012 (7) | 0.031 (8) | 0.034 (9) | -0.005 (6) | -0.006 (6) | -0.004 (7) |
| C10 | 0.008 (7) | 0.037 (9) | 0.032 (8) | -0.008 (6) | -0.001 (6) | -0.006 (7) |
| O1 | 0.025 (5) | 0.034 (6) | 0.029 (6) | 0.003 (5) | -0.005 (5) | -0.020 (5) |
| O2 | 0.064 (10) | 0.034 (7) | 0.043 (8) | 0.002 (7) | -0.005 (7) | -0.002 (6) |
| O3 | 0.032 (6) | 0.056 (8) | 0.025 (6) | -0.008 (6) | 0.000 (5) | -0.012 (6) |
| O4 | 0.020 (5) | 0.036 (6) | 0.024 (6) | -0.005 (4) | -0.003 (4) | -0.008 (5) |
| O5 | 0.036 (7) | 0.079 (11) | 0.076 (11) | -0.014 (7) | -0.012 (7) | -0.046 (9) |
| O6 | 0.029 (5) | 0.043 (7) | 0.037 (7) | -0.009 (5) | -0.006 (5) | -0.020 (5) |

Table S.7. Geometric parameters (Å, º) for **2**.

| Cd1—N2^i^ | 2.256 (12) | N4—O5 | 1.182 (19) |
| --- | --- | --- | --- |
| Cd1—N1 | 2.265 (12) | N4—O4 | 1.244 (16) |
| Cd1—O1 | 2.328 (11) | N4—O6 | 1.306 (17) |
| Cd1—O1^ii^ | 2.375 (10) | C1—C2 | 1.39 (2) |
| Cd1—O4 | 2.442 (10) | C2—C3 | 1.35 (2) |
| Cd1—O4^iii^ | 2.493 (11) | C3—C4 | 1.41 (2) |
| Cd1—O6^iii^ | 2.500 (11) | C3—C6 | 1.50 (2) |
| N1—C1 | 1.31 (2) | C4—C5 | 1.39 (2) |
| N1—C5 | 1.32 (2) | C6—C7 | 1.39 (2) |
| N2—C9 | 1.33 (2) | C6—C10 | 1.41 (2) |
| N2—C8 | 1.36 (2) | C7—C8 | 1.37 (2) |

Table S.7. (*Cont.)*

| N2—Cd1^iv^ | 2.256 (12) | C9—C10 | 1.36 (2) |
| --- | --- | --- | --- |
| N3—O2 | 1.22 (2) | O1—Cd1^ii^ | 2.375 (10) |
| N3—O3 | 1.230 (19) | O4—Cd1^iii^ | 2.493 (11) |
| N3—O1 | 1.328 (17) | O6—Cd1^iii^ | 2.500 (11) |
|  |  |  |  |
| N2^i^—Cd1—N1 | 175.8 (4) | O2—N3—O3 | 123.3 (15) |
| N2^i^—Cd1—O1 | 94.7 (5) | O2—N3—O1 | 117.9 (14) |
| N1—Cd1—O1 | 85.2 (5) | O3—N3—O1 | 118.7 (15) |
| N2^i^—Cd1—O1^ii^ | 90.4 (5) | O5—N4—O4 | 123.9 (13) |
| N1—Cd1—O1^ii^ | 93.6 (4) | O5—N4—O6 | 121.2 (13) |
| O1—Cd1—O1^ii^ | 73.4 (4) | O4—N4—O6 | 114.6 (12) |
| N2^i^—Cd1—O4 | 89.0 (4) | N1—C1—C2 | 121.4 (15) |
| N1—Cd1—O4 | 86.8 (4) | C3—C2—C1 | 121.0 (15) |
| O1—Cd1—O4 | 94.6 (4) | C2—C3—C4 | 117.5 (15) |
| O1^ii^—Cd1—O4 | 167.9 (4) | C2—C3—C6 | 123.3 (14) |
| N2^i^—Cd1—O4^iii^ | 90.2 (5) | C4—C3—C6 | 119.2 (15) |
| N1—Cd1—O4^iii^ | 88.5 (4) | C5—C4—C3 | 117.9 (15) |
| O1—Cd1—O4^iii^ | 159.9 (3) | N1—C5—C4 | 122.9 (15) |
| O1^ii^—Cd1—O4^iii^ | 126.1 (4) | C7—C6—C10 | 117.5 (14) |
| O4—Cd1—O4^iii^ | 66.0 (4) | C7—C6—C3 | 120.9 (14) |
| N2^i^—Cd1—O6^iii^ | 88.2 (5) | C10—C6—C3 | 121.5 (14) |
| N1—Cd1—O6^iii^ | 94.1 (5) | C8—C7—C6 | 118.9 (15) |
| O1—Cd1—O6^iii^ | 148.5 (4) | N2—C8—C7 | 123.4 (14) |
| O1^ii^—Cd1—O6^iii^ | 75.3 (4) | N2—C9—C10 | 123.8 (15) |
| O4—Cd1—O6^iii^ | 116.8 (4) | C9—C10—C6 | 119.1 (15) |
| O4^iii^—Cd1—O6^iii^ | 50.9 (3) | N3—O1—Cd1 | 125.2 (9) |
| C1—N1—C5 | 119.3 (14) | N3—O1—Cd1^ii^ | 125.8 (10) |
| C1—N1—Cd1 | 121.0 (11) | Cd1—O1—Cd1^ii^ | 106.6 (4) |
| C5—N1—Cd1 | 119.5 (10) | N4—O4—Cd1 | 147.0 (9) |
| C9—N2—C8 | 117.2 (13) | N4—O4—Cd1^iii^ | 98.0 (8) |
| C9—N2—Cd1^iv^ | 120.4 (11) | Cd1—O4—Cd1^iii^ | 114.0 (4) |
| C8—N2—Cd1^iv^ | 122.2 (10) | N4—O6—Cd1^iii^ | 95.9 (8) |

Symmetry codes: (i) *x*, *y*-1, *z*+1; (ii) -*x*, -*y*+1, -*z*+2; (iii) -*x*+1, -*y*+1, -*z*+2; (iv) *x*, *y*+1, *z*-1.
